# Salp blooms drive strong increases in passive carbon export in the Southern Ocean

**DOI:** 10.1038/s41467-022-35204-6

**Published:** 2023-02-02

**Authors:** Moira Décima, Michael R. Stukel, Scott D. Nodder, Andrés Gutiérrez-Rodríguez, Karen E. Selph, Adriana Lopes dos Santos, Karl Safi, Thomas B. Kelly, Fenella Deans, Sergio E. Morales, Federico Baltar, Mikel Latasa, Maxim Y. Gorbunov, Matt Pinkerton

**Affiliations:** 1 National Institute of Water and Atmospheric Research (NIWA), Hataitai, Wellington 6021 New Zealand; 2 Scripps Institution of Oceanography, University of California at San Diego, San Diego, CA 92093 USA; 3 Department of Earth, Ocean, and Atmospheric Science, Florida State University, Tallahassee, FL 32304 USA; 4 Center for Ocean-Atmospheric Prediction Studies, Florida State University, Tallahassee, FL 32310 USA; 5 Instituto Español de Oceanografía, Centro Oceanográfico de Gijón, Avenida Príncipe de Asturias, 70 bis, 33212 Gijón, Spain; 6 Department of Oceanography, University of Hawai’i at Mānoa, Honolulu, HI 96822 USA; 7 Asian School of the Environment, Nanyang Technological University, 50 Nanyang Avenue, Singapore, 639798 Singapore; 8 National Institute of Water and Atmospheric Research, P.O. Box 11-115, Hamilton, New Zealand; 9 College of Fisheries and Ocean Sciences, University of Alaska, Fairbanks, AK 99775 USA; 10 Department of Microbiology and Immunology, University of Otago, Dunedin, New Zealand; 11 Department of Functional & Evolutionary Ecology, University of Vienna, Vienna, 1090 Austria; 12 Environmental Biophysics and Molecular Ecology Program, Department of Marine and Coastal Sciences, Rutgers, The State University of New Jersey, New Brunswick, NJ 08901 USA

**Keywords:** Ecosystem ecology, Carbon cycle, Marine biology

## Abstract

The Southern Ocean contributes substantially to the global biological carbon pump (BCP). Salps in the Southern Ocean, in particular *Salpa thompsoni*, are important grazers that produce large, fast-sinking fecal pellets. Here, we quantify the salp bloom impacts on microbial dynamics and the BCP, by contrasting locations differing in salp bloom presence/absence. Salp blooms coincide with phytoplankton dominated by diatoms or prymnesiophytes, depending on water mass characteristics. Their grazing is comparable to microzooplankton during their early bloom, resulting in a decrease of ~1/3 of primary production, and negative phytoplankton rates of change are associated with all salp locations. Particle export in salp waters is always higher, ranging 2- to 8- fold (average 5-fold), compared to non-salp locations, exporting up to 46% of primary production out of the euphotic zone. BCP efficiency increases from 5 to 28% in salp areas, which is among the highest recorded in the global ocean.

## Abstract (web summary)

Gelatinous bloom-forming zooplankton—salps—alter microbial communities and quintuple the flux of sinking particles from the surface to the deep, strongly enhancing the ability of the ocean to sequester CO_2_.

## Introduction

The Southern Ocean covers ~1/3 of the world’s ocean and plays a fundamental role in global climate regulation^1,2^. The biological carbon pump (BCP), defined as ecosystem processes that uptake CO_2_ via photosynthesis and transport organic carbon to depth, has profound implications at planetary scales, with the architecture and efficiency of food webs playing an important role in the sequestration of carbon to deep waters. While distinct biomes within the Southern Ocean have variable efficiencies in BCP strength^3^, overall it accounts for a significant (2–3 Pg C y^−1^) fraction of the global biological pump (5–13 Pg C y^−1^)^4^. Predicting the response of the Southern Ocean BCP to climate change requires elucidation of ecological mechanisms that drive BCP variability^5^ in this important ocean area.

Salps are gelatinous zooplankton grazers that are widespread in the Southern Ocean^6^. Climate change-induced ocean warming may be allowing salps to expand into more southern waters in the vicinity of the Antarctic continent, where they can potentially displace krill^7^. The potential southward range expansion of salps may have important consequences for both the food web and the BCP, and biogeochemical models will benefit from a quantitative understanding of their effects on the marine carbon cycle. High particulate organic carbon (POC) export associated with salp blooms has been found in different environments including the Lazarev sea^8^ and Western Antarctic Peninsula (WAP)^9^ in the Southern Ocean, but also the Sargasso sea^10^, the northern Arabian sea^11^ and the California Current^12^. The combination of high ingestion rates^13^, extensive bloom formation^14^, ability to feed on a wide range of prey sizes (<1–1000 µm)^15,16^, and fast-sinking^17^ of large^9^ fecal pellets (FP) ultimately drive high rates of ocean carbon sequestration when salp blooms occur^18^. Modeling efforts to quantify salp contribution to export on a global scale are few but suggest substantial contributions to the BCP^19^ and support the need for further mechanistic studies that attempt to understand the conditions that give rise to salp blooms, as well as the temporal evolution of export related to a bloom cycle. Despite the examples cited above, studies quantifying how salp blooms alter carbon export budgets are quite limited—given that blooms often coincide with enhanced primary production (as food is required for population growth), it is not clear if salps substantially enhance export when otherwise this production would not be exported, or if they proliferate in conditions that will lead to high export independently. Disentangling these factors require simultaneous measurements of phytoplankton growth, micro- and mesozooplankton grazing, salp standing stocks, salp grazing or FP production, and carbon export using methods that integrate over the appropriate time scales (hours to days) in locations that have different environmental conditions—studies of this type are lacking. In addition, grazing by salps can alter the composition of both exported prey and the remaining assemblage, a process that is anticipated yet difficult to show. Salps are filter feeders, largely unselective in prey type except for size selection, consuming submicron-sized cells, pico-, nano-, and microplankton^13,16,20–25^. Thus, their grazing can also affect remineralization, as smaller-sized cells that would otherwise remain in the euphotic zone can be exported to depth altering community composition via changed sinking patterns. While the episodic nature of salp blooms has precluded controlled investigations in the past, in this study we successfully predicted the timing and location of salp blooms in the Southwest Pacific sector of the Southern Ocean, and present results from the first whole plankton food web process study quantifying salp-bloom impacts on BCP efficiency.

**Fig. 1:**
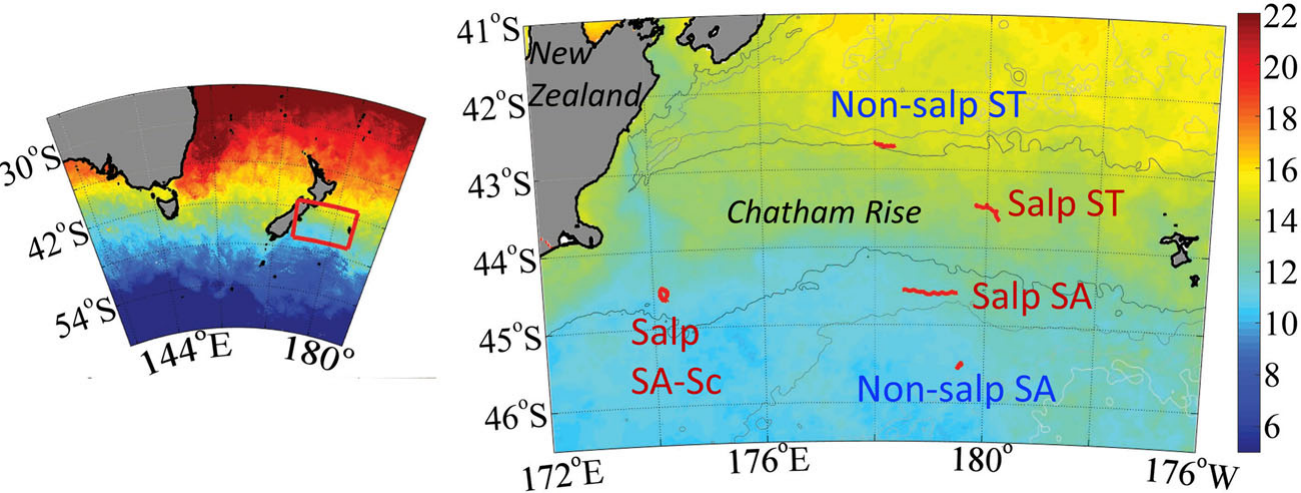
Study area. Lagrangian sampling (tracks shown as red lines/dots). Water mass types: Subantarctic Southland Current (SA-Sc), Subantarctic (SA), and Subtropical (ST). The location of the bathymetric feature is labeled “Chatham Rise” and extends to the east of the Chatham Islands, and the island of New Zealand is to the west of the study location. Colors indicate sea surface temperature (see color bar).

Our SalpPOOP (Salp Particle expOrt and Ocean Production) study takes place in Southern Ocean waters near the Chatham Rise (Fig. 1), where nitrogen-limited Subtropical (ST) and iron-limited Subantarctic (SA) surface water masses meet to form the productive Subtropical Front (STF)^26^. Results from fisheries trawls^27^, and predator diets^28–31^ suggest that salps are ecologically important in this region. Salp blooms are likely frequent and periodic occurrences in New Zealand (NZ) waters, as they are recurring prey for three species of deepwater oreos^31^, one species of sea perch^30^, two species of warehou^29^, NZ sea lions^28^, and Bryde’s whales^32^.

The goals of this study are: (i) to determine the effect of salp grazing on the microplankton community, (ii) to quantify the difference in export flux in bloom and non-bloom conditions, (iii) to determine if the enhanced BCP coincident with salp blooms is due to their FPs or the phytoplankton community that enables the salp bloom, and (iv) to investigate if salp blooms can alter the composition of prey exported out of the euphotic zone. We investigate flux patterns in five locations within ST and SA water masses (and with and without salp bloom in each). Our experimental design follows a Lagrangian framework, where sampling of water parcels is conducted over periods of 3–7.5 days (Table 1). Repeat measurements include hydrographic and nutrient conditions; salp abundance, biomass, and grazing; and phytoplankton stocks and growth/grazing rates. We also assess particle export flux using free-drifting Particle-Interceptor Traps (PIT) at different depths (typically 70, 100, 300, and 500 m) and ^238^U-^234Th^ disequilibrium methods^33^.

**Table 1:** SalpPOOP experiment cycle characteristics

Cycles in order sampled		Days	NPP (mg C m^−2^ d^−1^)	Areal chl *a* (mg m^−2^)	*S. thompsoni* oozooid abundance (m^−2^)	*S. thompsoni* blastozooids abundance (m^−2^)	*S. thompsoni* oozooid biomass (g C m^−2^)	*S. thompsoni* blastozooids biomass (g C m^−2^)	Other salps (salps m^−2^)	Other salp species
Salp SA-Sc	A	5	638 ± 174	39 ± 4.8	23 ± 8	213 ± 82	0.5 ± 0.2	0.9 ± 0.5	3 ± 3	*Soestia zonaria*
	B	2.5	390 ± 5	49 ± 5.1	6 ± 1	1120 ± 308	0.1 ± 0.0	0.7 ± 0.2	1 ± 0.5	*Soestia zonaria*
Salp SA		5.5	318 ± 67	28.1 ± 1.9	5 ± 2	124 ± 35	0.1 ± 0.0	0.3 ± 0.2	4.3 ± 2.2	*Thetys vagina, Pegea confederata*
Non-salp ST		3	746 ± 388	64.9 ± 9.6	0	0	0	0	6 ± 3	*Soestia zonaria, Salpa fusiformes, Ihlea magalanica, Thalia democratica*
Salp ST		4	452 ± 102	46.5 ± 12.3	3 ± 0.7	64 ± 15	0.02 ± 0.00	1.0 ± 0.2	2 ± 1	*Soestia zonaria, Salpa fusiformes, Ihlea magalanica, Thalia democratica*
Non-salp SA		3	233 ± 44	26.5 ± 10	0	0	0	0	1 ± 0.3	*Soestia zonaria*

Cycle duration, integrated net primary production (NPP) and chl *a* integrated for the euphotic zone (0.1% surface PAR), *S. thompsoni* abundance (m^−2^) and biomass (g C m^−2^) by stage (means ± SE). Salp SA-Sc had two deployments of Particle Interceptor Traps (PIT), so the cycle is divided into A and B time periods. Experiments are listed in the order they were sampled. Note that the most abundant salp was *S. thompsoni*, and the abundances of the “other salp species” are aggregated, since they were low, to indicate their contribution to both “salp” and “non-salp” locations.

## Results

### Subtropical and Subantarctic water mass characteristics and phytoplankton communities

Target study areas are identified based on the demersal habitat of New Zealand fishes that prey heavily on salps^29,31^. Lagrangian experiments (hereafter referred to as “cycles”) take place in five water parcels. This includes three SA cycles (one influenced by the Southland Current, which is a primarily (90%) SA current^34^ [designated as “SA-Sc”], and two in SA proper) and two ST cycles (Fig. 1). One cycle in each water mass is devoid of a salp bloom, for a quasi-controlled comparison with salp-bloom conditions in the same water mass. Temperature–salinity (T-S) plots generally support the classification of water parcels as SA or ST, although mixing is evident in the T-S characteristics of Salp SA-Sc and Salp ST sites (Supplementary Figs. 1 and 2).

**Fig. 2:**
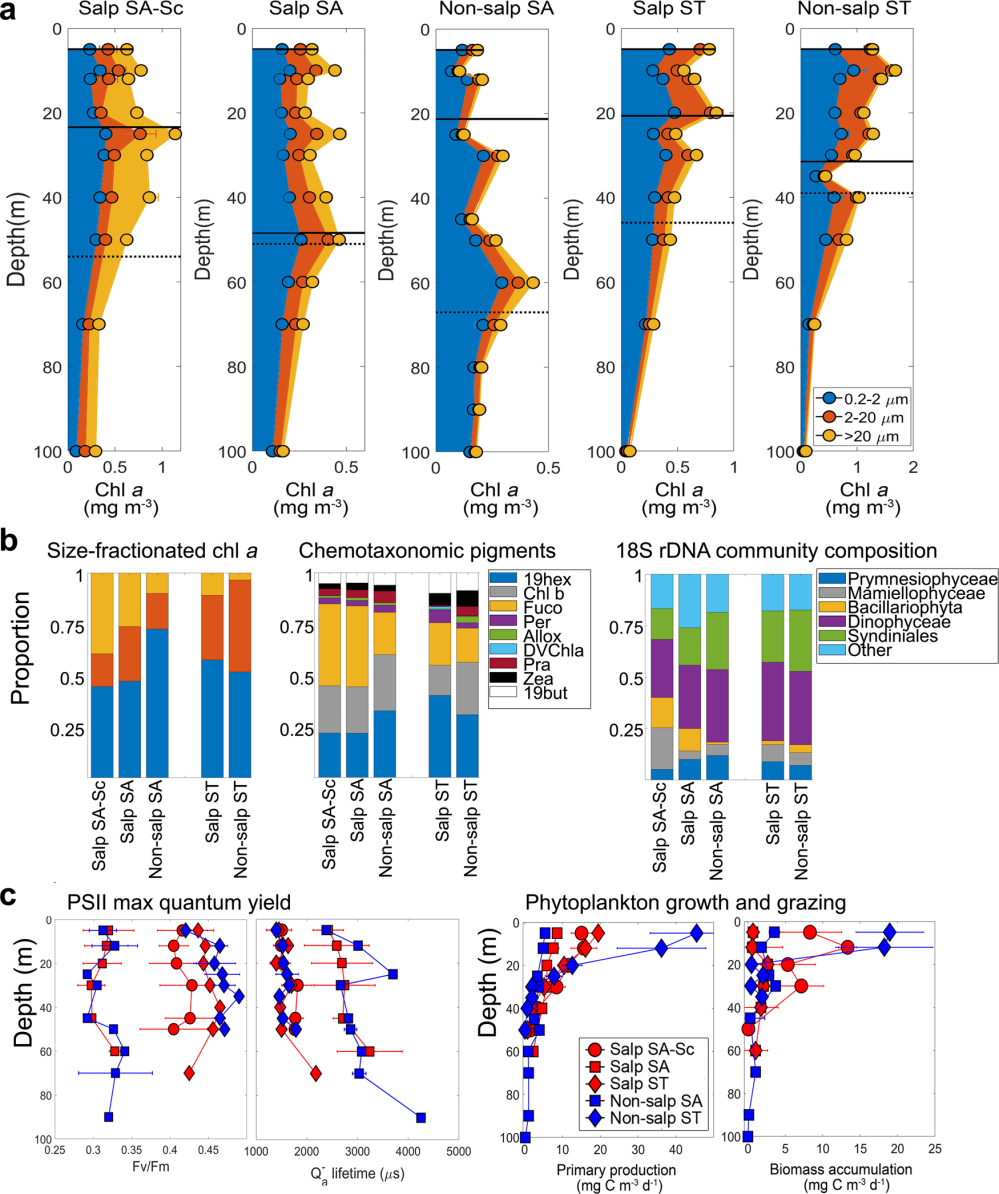
Phytoplankton community metrics. **a** Depth-resolved size-composition (0.2–2 µm, 2–20 µm, >20 µm) of chlorophyll *a* (see legend for colors) for each experimental cycle. Note different scales on *x* axis. Mixed layer is denoted by the straight line (shallower depth), and euphotic zone (0.1% PAR) by the dashed line (deeper depth). **b** Autotrophic integrated community composition: size-fractionated chlorophyll *a* (color-coded as in (**a**), pigment-based composition, and DNA-based composition, with colors corresponding to legend specifications. **c** PSII maximum quantum yield: phytoplankton physiology indicated by the Photosystem 2 (PSII) maximum quantum yield (Fv/Fm) and reoxidation kinetics (Q_a_^—^lifetime), and Phytoplankton growth and grazing: net primary productivity (NPP) and phytoplankton biomass accumulation (NPP—μzoo grazing). Errors are standard deviation. Symbols and colors are indicated in the legend: red markers indicate salp bloom locations, blue markers indicate non-salp locations, circles are for Subantarctic Southland Current (SA-Sc), squares are for Subantarctic (SA) waters, and diamonds are for Subtropical (ST) waters.

The phytoplankton community composition is generally consistent among the ST or SA parcels, but with important differences. Salp cycles in SA waters are characterized by the highest contribution of >20 µm cells, likely due to diatoms as indicated by Fucoxanthin and *Bacillariophyta* DNA reads (Fig. 2a, b and Supplementary Table 1). The Salp SA-Sc (first cycle) has higher phytoplankton biomass compared to the other SA locations, but not overall. The Non-salp SA cycle shares similar phytoplankton biomass to Salp SA, but contrastingly the 0.2–2 µm size class dominates (Fig. 2b). The ST cycles, salp and non-salp, share greater similarity compared to SA locations, with low contributions of >20 µm cells, greater contributions of nanophytoplankton (2–20 µm), and a community dominated by *Prymnesiophyceae*. The highest phytoplankton biomass of all locations is found at the non-salp ST location (Fig. 2a, b). Dominance by prymnesiophytes in ST locations is indicated by the markers 19-hexanoyloxyfucoxanthin and 19-butanoyloxyfucoxanthin, and 18S sequence data (Fig. 2b and Supplementary Table 1).

Phytoplankton physiology measured using variable fluorescence and the kinetics of photosystem II (PSII) reaction centers suggests iron stress in phytoplankton inhabiting SA waters. However, Salp SA-Sc phytoplankton physiology conditions are similar to ST, indicating that frontal mixing alleviates iron stress. Consequently, Salp SA-Sc is the one location with relatively high nitrate surface concentrations (1–2 µM) not Fe-limited, sustaining high diatom biomass (Fig. 2b). Highest net primary production (NPP) is in Non-salp ST waters, and lowest in Non-salp SA (Table 1 and Fig. 2c). We quantify biomass accumulation of phytoplankton, which is the amount that is not grazed by microzooplankton and can thus either accumulate in the euphotic zone, be grazed by zooplankton, or be exported via phytoplankton direct sinking. This biomass accumulation is also highest in Non-salp ST waters, followed by Salp SA-Sc, while the other three locations have values of similar magnitude (Fig. 2c).

### Salp abundance and size structure, bloom evolution, and fecal production rates

**Fig. 3:**
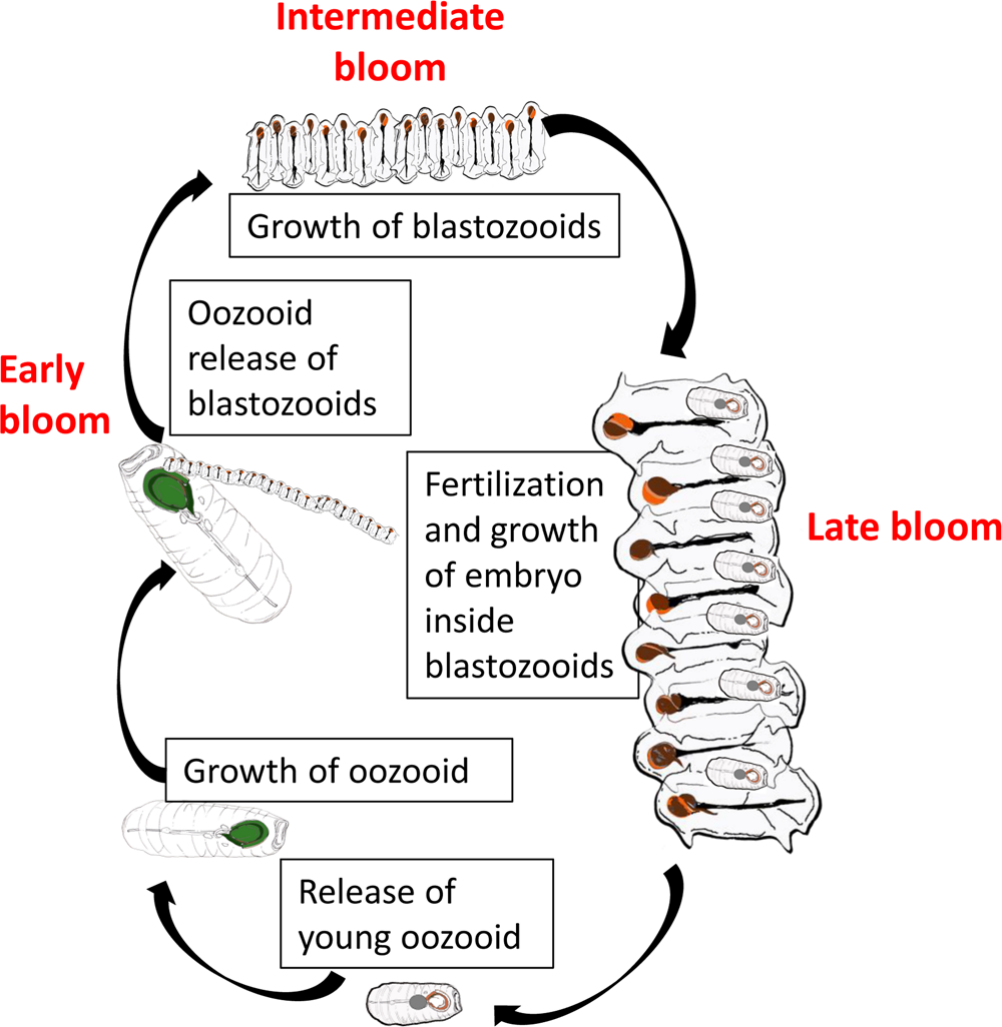
Simplified schematic of the life cycle of salps. The oozooid refers to the asexual (or solitary) stage. The oozooid begins to produce chains of blastozooid clones (also known as the sexual or aggregate phase) typically in response to enhanced phytoplankton biomass, triggering the onset of a bloom. Once blastozooids reach maturity they each harbors one oozooid embryo, which is birthed live. After releasing the embryo, the female blastozooids mature their male testis (not shown here), and the cycle is completed with the maturation of the young oozooid. The length of the entirety of the cycle for *S. thompsoni* in Antarctic waters (−1–2 °C) has been estimated to range between 2 and 9 months depending on the study, although the generation time of the blastozooids is much shorter compared to oozooids. Growth rates of blastozooids during SalpPOOP suggest a shorter life-cycle duration in the warmer waters (~10 °C) of the Chatham Rise, ranging between 12 and 42 days (average 23). The blastozooids emerge as <1 mm sized animals and continue to grow to ~50 mm when mature females.

**Fig. 4:**
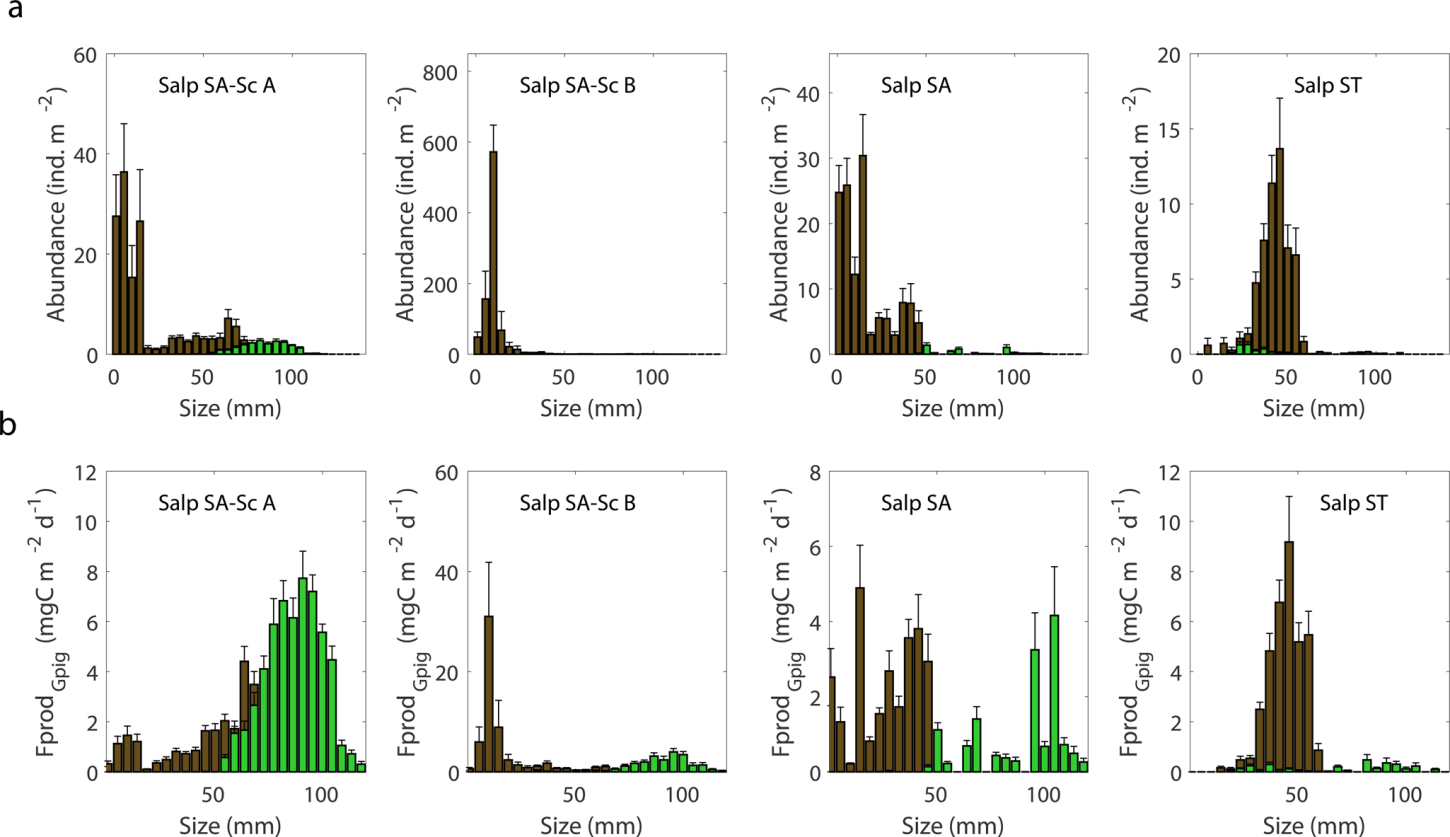
Size-specific salp abundance and fecal pellet production rates. **a** Abundance (day/night averaged) of salps in the upper 200 m, and **b** fecal pellet production (Fprod_Gpig_) rates by size class for the four salp locations. Green bars correspond to oozooids and brown bars correspond to blastozooids. Error bars for all panels are SE.

*S. thompsoni* dominates the salp composition during this study, with distinctly different age and stage structures in different locations. While our intent was to sample salp blooms in waters with different characteristics, which we did, our cycles also follow a temporal progression in bloom evolution following the predominant currents moving from the New Zealand coast (Fig. 1), such that each location also has a very different size- and stage- structured bloom. A schematic of the life cycle of salps (*S. thompsoni*^35^) is depicted in Fig. 3, which details the transition between the asexual (oozooid) and sexual (blastozooid) stages of the organism as the bloom evolves. Generation times of the different stages depends on the study and location^36–38^, but in our study the blastozooid maturation was estimated to range between 12 and 42 (average 23) days^38^. Figure 4a summarizes the salp abundance and size structure by location. The Salp SA-Sc cycle lasted 7.5 days, although Particle Interceptor Traps (PIT) were recovered on day 5 and re-deployed for an additional 2.5 days, such that we divide the experiment into two periods (A, B). The population structure is characteristic of an early-stage bloom. The highest abundances of salps are associated with this location, including the highest abundances of large oozooids during Period A: mean (±std) = 22 ± 8 ind. m^−2^ (max = 110 ind. m^−2^), which decreased over the duration of the experimental cycle (Supplementary Figs. 3a, 4 and Table 1). A substantial change in blastozooids is also documented: large, mature individuals present during Period A, with average size of individuals = 39 mm (±3 mm) in the first couple of days of sampling and cycle averages of 213 ± 82 ind. m^−2^, are replaced with chains of small blastozooids as the bloom evolves, averaging 1120 ± 308 ind. m^−2^ in Period B (Supplementary Figs. 3a and 5). An intermediate bloom stage is inferred for Salp SA waters, with low large oozooid abundance (5 ± 2 ind. m^−2^) as a result of low abundances of large mature oozooids and an absence of young embryos not yet released into the water column (Supplementary Fig. 3b). A mixture of both large and small animals (range 7–32 mm) of intermediate abundances (Supplementary Fig. 6) results in the relatively small average length (13 ± 7 mm) that characterizes the Salp SA blastozooid cohort. Finally, larger blastozooids (32 ± 8 mm) and younger, smaller oozooids characterize the population in Salp ST (Supplementary Figs. 3c and 7). The smaller oozooids, with relatively low abundances (3 ± 0.7 ind. m^−2^), still carried remnants of the maternal placenta. The release of the new generation of asexual individuals marks the completion of their reproductive cycle (Fig. 3)^35,38^. Although salps were detected in the non-salp locations, these were present in low abundances and also included species other than *S. thompsoni* (Table 1).

FP production, e.g., egestion, is calculated using two methodologies—one based on grazing estimated from phytoplankton pigments in individuals collected during the cycles (Fprod_Gpig_) and an egestion efficiency of 0.36^39^, and the other using a published length-FP production relationship (Fprod_Iversen_) derived for Antarctic waters^40^. Both methods are scaled to the temperature of this study using a Q10 of 2 (see “Methods” for details), with results overall similar in magnitude (Supplementary Figs. 4–7) although generally higher values using Fprod_Iversen_ compared to Fprod_Gpig_ (Supplementary Table 4). FP production is highest in Salp SA-Sc A and B, although the hypothesized size distribution of pellets, expected from the size distribution of the animals, varies noticeably (Fig. 4b). Oozooid FP production only contributes substantially to the total egestion in the early phase of Salp SA-Sc (Period A): 40–70% of the total egestion (depending on the method used). Egestion of salps in Salp SA-Sc is 1.6– 2.3-fold the total POC flux at 200 m (depending on Fprod_Gpig_ or Fprod_Iversen_, respectively). For Salp SA and Salp ST, the total FP production is equivalent to 50–70% of the total POC flux at 200 m, depending on location and methodology used. The size distribution of the salp population suggests that large fast-sinking pellets dominate flux during Salp SA-Sc A and Salp ST, while Salp SA-Sc B has greater contributions of smaller fecal pellets and Salp SA is more uniform (Fig. 4b and Supplementary Fig. 6).

### Growth and grazing balances

The balance of NPP, microzooplankton grazing, mesozooplankton grazing, and salp grazing suggests that the net rate of change for phytoplankton in our study is generally negative, with the exception of Non-salp ST, which has positive net growth (Supplementary Fig. 8). While NPP rates are similar between Non-salp ST (746 ± 102 mg C m^−2^ d^−1^) and Salp SA-Sc A (681 ± 174 mg C m^−2^ d^−1^), loss terms of the latter include higher mesozooplankton grazing and substantially higher salp grazing, which despite microzooplankton exerting lower grazing control, results in a negative rate of change. Microzooplankton grazing rates are relatively similar among salp locations, such that the magnitude of NPP decrease per day is set by the combination of salp and zooplankton grazing. Interestingly, the sum of growth and grazing for the Non-salp SA cycle also results in a negative rate of change, likely driven by low NPP relative to microzooplankton grazing.

### Carbon export and salp fecal pellet contribution to the BCP

**Fig. 5:**
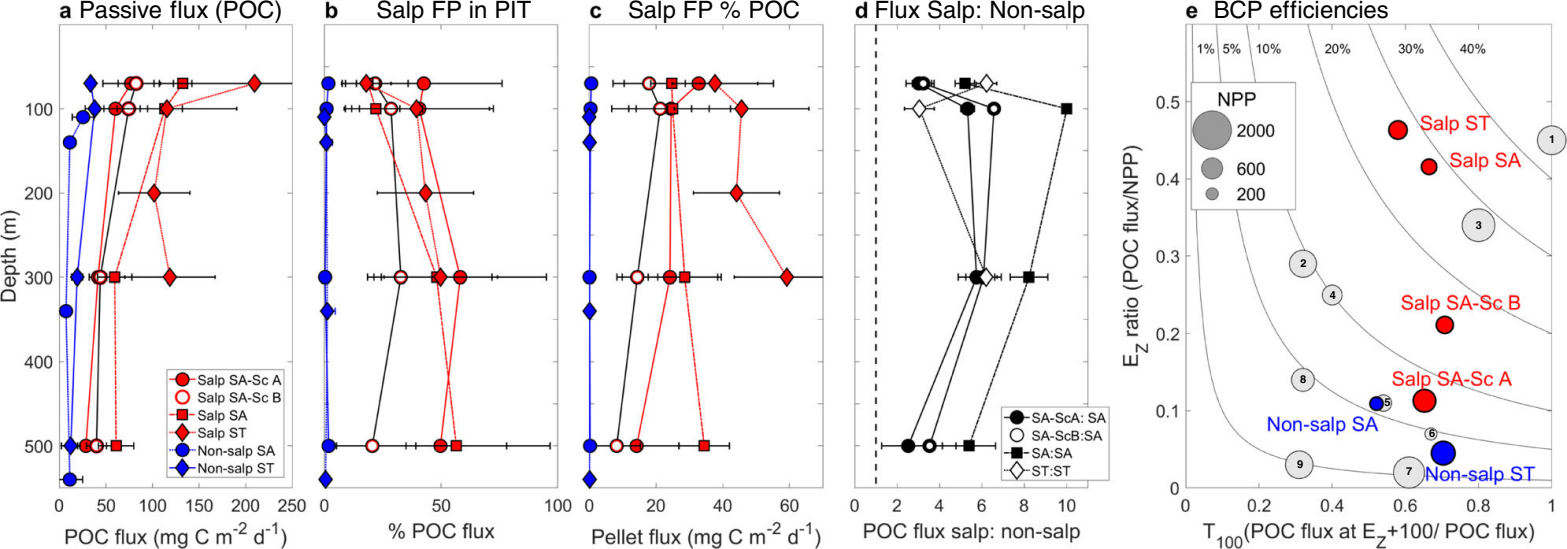
Patterns in carbon export flux. Mean ± std of **a** export fluxes of particulate organic carbon (POC), **b** carbon flux due to recognizable salp fecal pellets (FP), **c** relative contribution of intact salp FP to POC flux. Colors and symbols for experimental cycles are denoted in the legend in (**a**). **d** Ratio of POC flux between Salp and non-salp locations, with three comparisons for SA waters, and one for ST waters. Doted line indicates a ratio of 1. **e** E_Z_ ratio, the ratio of POC flux: net primary production (NPP), as a function of T_100_ (POC flux at E_Z_ + 100 m/POC flux). Numbers indicate locations compared in ref. 3: 1—North Atlantic Bloom Experiment (NABE) (spring, temperate North Atlantic), 2—Kiwi 7, 3—Kiwi 8 (Polar Front, Pacific sector, Southern Ocean), 4—K2 - D1 (subarctic NW Pacific), 5—K2 - D2 (subarctic, NW Pacific), 6—ALOHA (subtropical, central North Pacific), 7—EqPac (tropical, central Pacific), 8—OSP – Aug (summer, NE Pacific), 9—OSP – May (spring, NE Pacific). Circles are proportional to magnitude of NPP (see legend insert). Results from this study are in color: blue indicates non-salp locations, red indicates salp cycles during the SalpPOOP experiment.

Sinking POC measured using sediment traps indicates salp locations had significantly higher export flux compared to non-salp areas (Wilcoxon signed-rank test, paired difference test, *P* = 0.01, *n* = 24, rank-sum = 243) (Fig. 5a). During salp cycles, POC fluxes below the euphotic zone (70 m) range from 80 to 210 mg C m^−2^ d^−1^ (Salp SA = 80–130 mg C m^−2^ d^−1^, Salp ST = 210 mg C m^−2^ d^−1^). Flux into the mesopelagic zone at 300-m depth ranges from 42–60 mg C m^−2^ d^−1^ (SA) to 119 mg C m^−2^ d^−1^ (ST). POC export in non-salp areas is substantially lower, 25–33 mg C m^−2^ d^−1^ in the epipelagic (SA-ST), with mesopelagic flux (300 m) values about 5-fold lower than salp areas: 7 ± 6 mg C m^−2^ d^−1^ (SA) and 19 ± 6 mg C m^−2^ d^−1^ (ST) (mean ± std). Microscopical analyses of trap contents indicates that 20–40% of export at the base of the euphotic is attributable to intact, recognizable salp FPs (Fig. 5b, c), and salp pellets account for up to 50% of the mesopelagic (300–500 m) flux (Fig. 5c). The ratio of total POC flux in salp:non-salp areas is variable by location and depth, but ranges between twofold and tenfold, on average ~fivefold higher in salp areas (Fig. 5d).

The efficiency of the BCP is the product of the proportion of NPP that sinks out of the euphotic zone (E_Z_) and the proportion of this flux that sinks an additional 100 m into the mesopelagic zone (T_100_, the flux transmission^3^). The E_Z_ ratio is highly enhanced in areas with salps compared to non-salps (Fig. 5e and Supplementary Table 2), and also apparently increases with the stage of the salp bloom. In ST waters, the E_Z_ ratio is 0.05 without salps and increases to 0.46 in the location with salps (i.e., 46% of NPP exported below the euphotic zone); while in SA waters it increases from 0.11 to 0.42 when salps are present. Interestingly, the Ez ratio is similar (0.11) for Non-salp SA and the early bloom stage, Salp SA-Sc (Period A). The T_100_, however, is higher when salps are present, 0.52 (non-salp) vs 0.65 (early salp bloom, Salp SA-Sc A), increasing to 0.7 for the second period of Salp SA-Sc (B) as the Ez also increases (Fig. 5d). BCP efficiencies vary among locations as well, with only slightly higher values for the early period of Salp SA-Sc (A) compared to Non-salp SA (7.4% vs 5.6%), but increasing to 15% over the following 2.5 days (B). Both SA and ST salp locations share high BCP efficiencies (~27% of NPP is exported to 100 m below the euphotic zone) and are among the highest efficiencies recorded in the global ocean^41^.

**Fig. 6:**
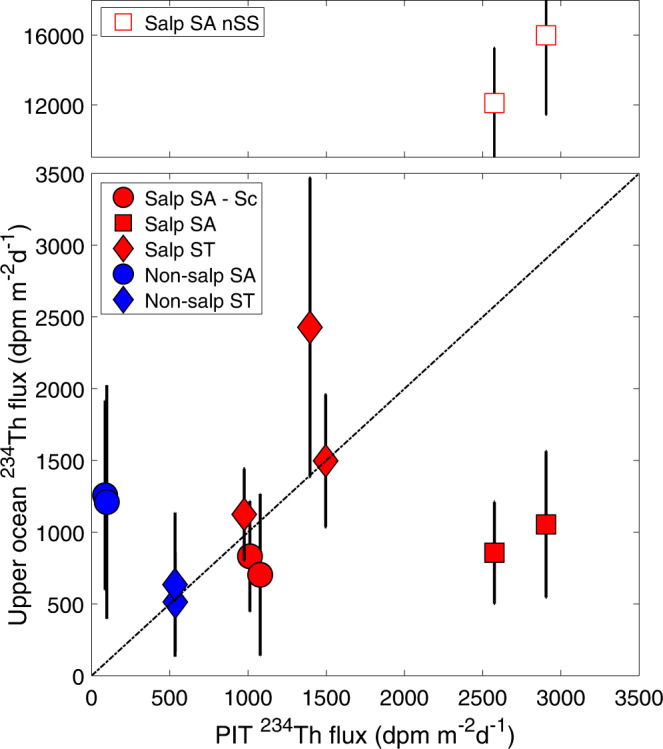
^234^Th flux out of the upper ocean. *x* axis indicates Th-234 flux estimates measured in PIT, while *y* axis indicates Th-234 flux estimates using the ^238^U:^234^Th disequilibrium method, using water profiles of Th-234 and salinity-derived U-238 estimates, with integration depths matching the depth of the Particle Interceptor Traps (PIT). Filled markers indicate values estimated using steady-state assumptions, open markers (Salp Subantarctic, Salp SA) indicate non-steady state assumptions (box above break). Note scale change for the non-steady state. Error bars are 1 std propagated from all uncertainty terms.

POC flux estimates based on ^238^U-^234^Th show high deficiencies in the surface, and throughout the water column for some casts conducted during the salp cycles (Supplementary Fig. 9). The deficiency during Salp SA-Sc is most pronounced in the upper 50 m. Salp SA has a large difference in estimates between the first and last day of the cycle, suggesting a lack of steady state due to increasing carbon export over time. Salp ST shows high deficiencies throughout the water column, even down to 300 m. Deficiencies in the non-salp cycles are generally restricted to the water column shallower than 150 m. A comparison of results between the ^238^U-^234^Th disequilibrium methodology and PIT flux shows general agreement with three of the five cycles when compared to ^234^Th flux measured in the PIT (Fig. 6). We calculated integrated rates using a steady state (SS) and a non-steady state (non-SS) model for Salp SA, given the different profiles sampled. Estimates using SS are an order of magnitude lower compared to non-SS, and neither estimate shows good agreement with the PIT flux, which is ~4 times higher than SS and ~5 times lower than non-SS deficiency. In addition, Non-salp SA shows disproportionally higher ^238^U-^234^Th-based export compared to PIT (~1 order of magnitude higher), which is likely due to a prior high-flux event that depleted ^234^Th from the water column.

Comparisons of the FP production and salp pellets at 200 m indicate that ~30% of the FPs can sink directly and are thus identifiable as intact pellets in Salp SA-Sc A. This percentage is slightly lower in the second portion of the cycle, when small blastozooids dominate (Supplementary Table 3). Approximately 60% of the estimated egestion (FPs) during Salp SA are identifiable as intact pellets in sediment traps, and pellets accounting for ≥100% of the estimated egestion (based on Fprod_Gpig_) are found in Salp ST at all depths below the euphotic zone. The range across the study area depends on the method: 24–112% Fprod_Gpig_ and 14–57% Fprod_Iversen_ (because FP production with this method was sometimes 50% higher) sink below 200 m.

### Microplankton community composition in the water column and PIT

**Fig. 7:**
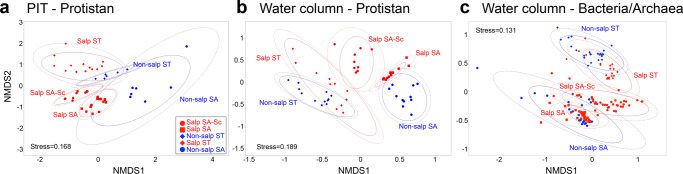
Non-metric multidimensional scaling (NMDS) of microbial communities during SalpPOOP. **a** Protistan communities sampled in Particle Interceptor Traps (PIT), **b** Protistan communities in the euphotic zone of the water column (WC) sampled from the conductivity–temperature-depth CTD rosette, and **c** Bacteria/Archaea communities in the euphotic zone of the water column (also sampled from the CTD). Water mass explained most of the variance among communities in the different locations—PIT protists: 7% variance, WC protists: 19%, WC prokaryotes: 20% (PERMANOVA, all *P* values «0.001). Salp/non-salp followed for protists (PIT: 4% variance, WC: 9% variance), and was also significant for prokaryotes (7.6% variability). Depth of collection explained similar levels of variability in protistan communities (PIT: 3%, WC: 8%), and was more important for prokaryotic communities (16% variance).

The effect of microbial communities in regulating export was investigated through community genetic analyses of three different groupings: PIT protists, water column (WC) protists, and WC prokaryotes. Variability between communities can be explained primarily by water mass (PIT = 7%, Protistan = 19%, and Bacteria/Archaea = 20% variance), but salp/non-salp delineations also explain 3% of variability in PIT and 8% in WC samples (Fig. 7). Composition of sinking phytoplankton (PIT) does not suggest that any one of the main algal groups is responsible for driving export across high export (salp) locations. Diatoms (*Bacillariophyta*) and coccolithophores (*Prymnesiophytes*) can drive export flux due to their dense mineral shells, but neither of these groups are present in noticeably higher contributions in PIT compared to WC (Supplementary Fig. 10a), and none of these groups are statistically correlated with high export fluxes (Supplementary Table 4). When analyzing the WC and PIT community together, the main driver of variability is method of collection (CTD or PIT) (not shown).

To investigate the possibility that salp grazing enhances export of specific phytoplankton types/species, we assessed the similarity between WC and PIT composition. This was done by quantifying the number of amplicon sequence variants (ASVs) present in statistically significantly different abundances between WC and PIT (DESeq2, R package^42^). A *Χ*^2^ test comparing not significant/significant ASVs between salp and non-salp locations (Supplementary Table 5), shows that in both the Salp SA-Sc and Salp ST location, there is greater similarity between the water column and PIT compared to the controls (non-salp areas). Interestingly, we do not detect any statistical difference in the similarity between Salp SA and Non-salp SA sequences. A further investigation into which species of phytoplankton are preferentially exported in the salp cycles only shows a clear result for ST waters. Both salp and non-salp ST waters have similar phytoplankton compositions, yet prymnesiophytes were only exported in considerable quantities in the Salp ST location, and these were among the sequences that were similar for both WC and PIT (Supplementary Fig. 10b). These sequences correspond primarily to *Gephyrocapsa oceanica*, a coccolithophore of ~10 µm size, which is present in both cycles, and the dominant prymnesiophyte in PIT at the Salp ST location. Exported particles in Non-salp ST waters do not show notable prymnesiophyte DNA contributions (less than 20 reads per sample). A comparison for SA waters is not so straightforward because the starting microplankton compositions between “salp” and “control” sites are quite different.

### Salp DNA in the environment

**Fig. 8:**
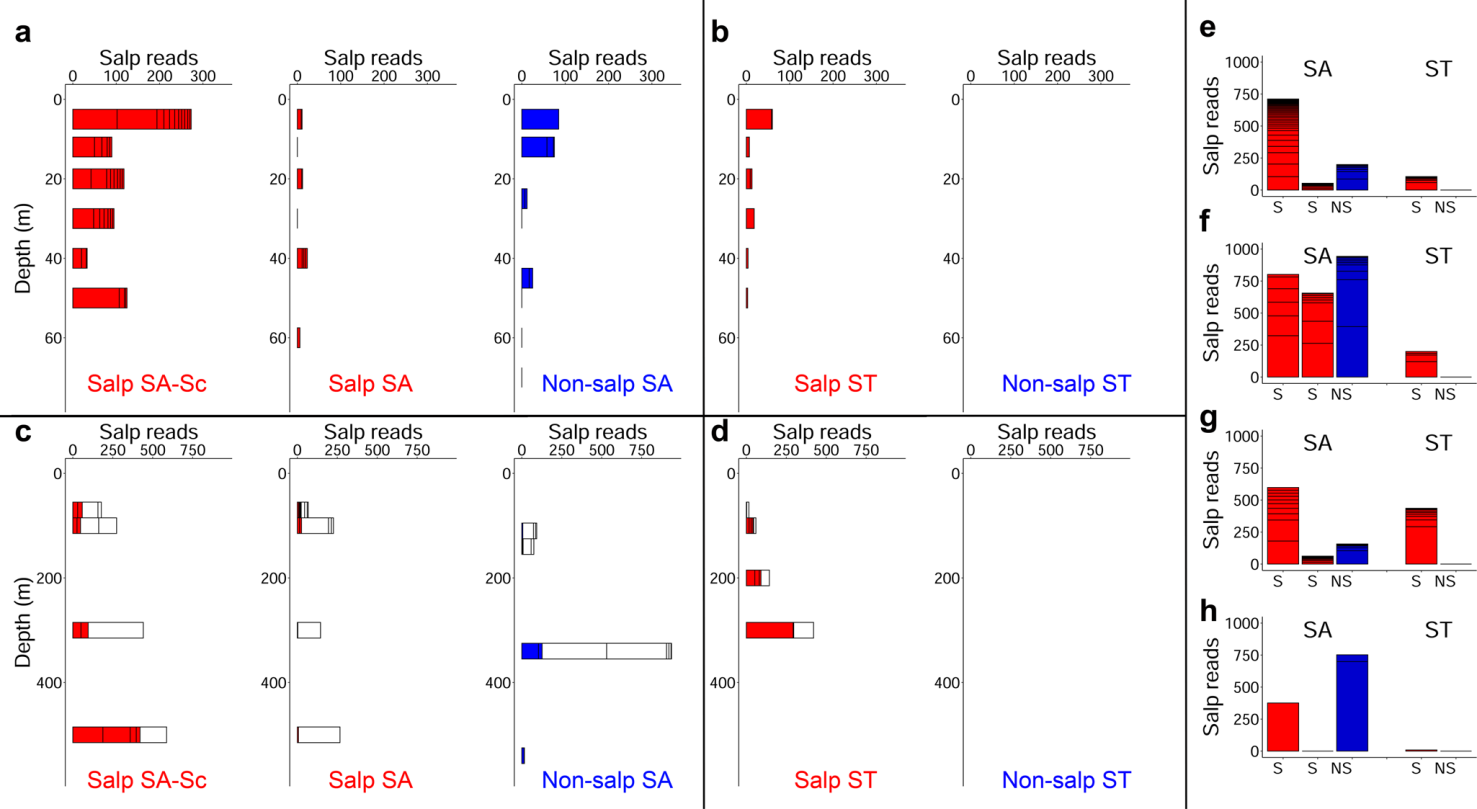
Salp reads in the water column, sinking particles, and sediments on the seafloor. Salp reads in the euphotic zone for **a** Subantarctic (SA) waters, and **b** Subtropical (ST) waters. Number of reads in Particle Interceptor traps (filled) and in situ pump (white) bars in **c** SA waters and **d** ST waters. Integrated amplicon sequence variant (ASV) reads in **e** water column, euphotic zone, **f** water column, euphotic and mesopelagic zones, **g** all traps combined, and **h** sediments on seabed. S, S, and NS in SA locations indicate: Salp Southland Current (SA-Sc), Salp SA, and Non-salp SA. S, NS in ST locations indicate: Salp ST, and Non-salp ST.

We further investigate the influence of salps in the environment by assessing ASVs of salps in the euphotic zone (WC, CTD), in the whole water column (In situ pump, ISP), in PIT, and in sediments on the seafloor. Salp DNA is detected in four of the five cycles—with the only exception in ST waters without salps (Fig. 8a, b, c, d). Read numbers are highest in Salp SA-Sc, across all sampling types, most noticeably in the euphotic zone (Fig. 8a, e). Interestingly, Salp SA water show very low reads in the euphotic zone, while Non-salp SA show higher numbers of reads compared to both Salp SA and Salp ST. ASVs in PIT are present in the mesopelagic samples for Salp SA-Sc, Non-salp SA, and Salp ST (Fig. 8c, d, g). Again, Salp SA shows low ASV reads in PIT (and mostly in the surface). However, the ISP-collected particles from Salp SA show read counts comparable to the other locations with salp DNA in the water (Fig. 8c, d, f). Finally, the sediments underlying the water column in the Non-salp SA location show the highest number of salp ASVs, followed by Salp SA-Sc (Fig. 8h), with detectable but marginal counts in Salp ST sediments (despite this location being the shallowest).

## Discussion

Our results demonstrate a pronounced effect of salp blooms on microbial dynamics and passive POC export flux in the region. In this discussion, we address the four goals outlined in the introduction, as well as the limitations and caveats from this study.

We determined that salp grazing had large and measurable effects on the phytoplankton net rate of change, with decreasing rates of NPP over the cycle duration in all salp locations, in part driven by decreasing standing stocks. As the phytoplankton in this region is generally kept under tight control by the microzooplankton in all seasons^43–45^, it is perhaps unsurprising that the addition of salp grazing tips the balance towards a negative rate of change. However, microbial dynamics in this region are different in ST vs SA waters. Past studies show microzooplankton grazing on phytoplankton in ST waters in the spring consumed ~70% PP, while SA waters are characterized by >100% of PP grazed by microzooplankton^43^. Our study is consistent with these results, where the Non-salp ST location shows a positive phytoplankton growth rate and a microzooplankton consumption rate equal to 69% of PP, and the Non-salp SA location shows a negative phytoplankton growth rate with 120% of PP consumed by microzooplankton. The effect of the salp bloom on the fate of primary production in ST waters shifts it from net growth to net loss, while in SA waters it leads to a faster decrease in phytoplankton standing stocks. In the early Salp SA-Sc location, the addition of salp grazing drives the ~1/3 decrease in NPP between Period A and B. Microzooplankton and non-salp mesozooplankton grazing in this location are roughly equivalent to the daily PP, and in the absence of grazing by salps, phytoplankton biomass would likely be in steady state. Thus, even moderate salp abundances lead to important reductions in phytoplankton biomass, especially in the Southwest Pacific which is characterized by relatively low PP and tight microbial coupling^45,46^.

We cannot directly answer the question of what types of plankton prey enable the formation of a salp bloom, because this study was not designed to determine the causality of the relationship between salp blooms and microbial community composition. However, the significance of the link between salp bloom waters with both eukaryotic and prokaryotic communities warrants future study. Incubations conducted during SalpPOOP indicate clear differences in size-selectivity by salp stage/size, where only the small blastozooids obtain substantial nutrition from small bacteria-sized particles, and large oozooids/blastozooids only efficiently retain cells >6 µm^16^. The variance in salp sizes across cycles suggest sizable impacts on the bacterioplankton primarily during Salp SA-Sc B, and greater effects on larger microplankton during Salp SA-Sc A and Salp ST. That the bloom is present in waters dominated by large diatoms (early bloom, western end of the Chatham Rise) and in waters dominated by prymnesiophytes (late bloom, ST waters eastward of bloom initiation) suggests that a specific taxonomic group of phytoplankton is not required for sustaining salp blooms, although it is unclear if both groups are equal in supporting salps or triggering bloom initiation. It is hard to compare the grazing rates among locations, given the differences in size and abundance. However, if we take the Fprod_Iversen_ to represent grazing that is independent of phytoplankton composition (since it depends only on salp length and temperature) and Fprod_Gpig_ to incorporate local conditions (since it is based on pigments from animals collected in situ, and temperature), we find that in Salp ST (where prymnesiophytes dominated) Fprod_Gpig_ is 0.5 Fprod_Iversen_, compared to 0.7–0.87 in SA waters, suggesting higher grazing rates in a diatom-dominated community. Further disentangling the effect of water mass characteristics, mixing in the frontal region, and grazing by salps on microbial dynamics requires sampling of similar-staged blooms with different initial conditions.

We conclude that the salp bloom is the main driver of the large shifts in regional POC export fluxes and BCP efficiency, given the lack of a correlation with nutrient and plankton variables such as NPP or phytoplankton size that are generally understood to drive export flux in the ocean. We estimate the differences in export flux between salp/non-salp locations to range between twofold and eightfold, with a mean of fivefold. Our interpretation that most of this enhanced flux is due to salp fecal pellets—either directly sinking or contributing to export downstream after disaggregation—is also consistent with past studies in the region^47,48^. While the lowest export fluxes in our study, in the non-salp locations, range between 5 and 10% of PP, past studies in these waters using floating PITs found export below the euphotic zone in the spring to be even lower, ranging between 0.5 and 1.4% of PP^47^. Long-term studies indicate the region typically has low export fluxes, with some episodic high-flux events^48^. Given the high variability of phytoplankton bloom and export processes, it is more appropriate for us to compare fluxes across locations within our own study. Conclusively determining the contribution of salp pellets is difficult because small pellets become fragmented and sink slowly. The only other study (to our knowledge) investigating *S. thompsoni* FP attenuation between 100 and 300 m^40^ also found values that were highly variable yet similar to ours. Approximately 20–100% of salp FPs measured at 100 m sank to traps at 300 m (mean 58% ± 40%^40^), when animals were small (<20 mm) and present in lower abundances. These values are in the same range as ours, 19–112% (Fprod_Gpig_) or 12–59% (Fprod_Iversen_), with the lowest values during Salp SA-Sc B when small blastozooids are dominant. High degradation is consistent with the observation that from Salp SA-Sc A to B, flux from recognizable salp pellets decreases. Our most conservative estimation of the BCP enhancement due to salps can be thus derived by comparing passive POC flux in non-salp locations to intact salp pellet flux from salp locations, by water mass. Comparing these values at 300 m, a depth below the euphotic zone and below which salp contribution ceases to vary, we find that the BCP is enhanced 2–4-fold when only intact sinking pellets are taken into account.

**Fig. 9:**
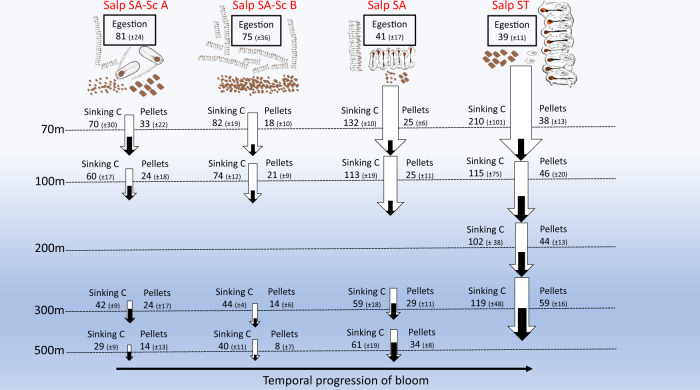
Schematic of salp composition, egestion, and export flux over the temporal evolution of the bloom. Salps, pellets, and arrows representative of abundance, size, and flux magnitude. Values are in mg C m^−2^ d^−1^, mean ± SE for Egestion (Fprod_Gpig_), mean ± std for Sinking C (particulate organic carbon, POC) flux. Depth horizons indicated by dotted lines are not drawn to scale. White arrows are the total POC flux at each depth, black arrows inside the white are the measured C flux of intact salp fecal pellets at each depth (both collected in particle intercept traps). Egestion is the sum for both blastozooids and oozooids in each location.

We interpret the enhanced POC export in the later salp cycles (relative to Salp SA-Sc) as including at least portions of the egestion produced in the earlier stages of the bloom, on the western end of the Chatham Rise advected eastward following the convergence front. We hypothesize that the Salp ST waters (the last salp cycle sampled), with the most advanced bloom encountered during SalpPOOP and the highest export flux, represent an endpoint of the temporal bloom evolution and decoupling between sinking POC and salp abundance and grazing. This suggested relationship between salp bloom stage (abundance and size distribution) and export among the different cycles is depicted in Fig. 9. The advection of the excess of material egested from nearshore to offshore, while hypothesized, is further supported by ^234^Th disequilibrium measurements. The ^238^U:^234^Th method calculates the deficiency of the daughter isotope ^234^Th relative to ^238^U—with a half-life of 24 days—which means this method integrates over a longer time period (~1 month) than sediment traps (~3.5–7 days for PIT, depending on deployment). This longer integration time is important in interpreting results based on ^234^Th, but also for deciding if a steady-state or non-steady-state model should be used to calculate export^33^. In the case of Salp SA, the two profiles show clear differences between the start and end of the cycle, indicating the system was not in a steady-state and export flux increased over this time period. We present results for Salp SA based on steady-state and non-steady-state assumptions but note that high temporal variability adds substantial uncertainty to these calculations. However, these results clearly indicate a dynamic system in which POC flux has been increasing over time. In the specific case of Salp ST waters, the higher export calculated from ^234^Th (compared to in PIT) in the deeper depths (200 and 300 m) suggests these waters experienced high sinking fluxes prior to the PIT deployment that disproportionally depleted ^234^Th from the water (i.e., ^234^Th is integrating the export signals seen during Salp SA-Sc and Salp SA, along with Salp ST). This observation, combined with slightly higher export flux at 300 m (relative to 100–200 m) from PIT, suggests that mesopelagic waters contained the signal (depleted ^234^Th) of high export upstream, as well as sinking particles not originating from the overlying surface waters. These could be slower sinking particles from the early bloom (likely disaggregated fecal pellets), but it is also possible that some of this organic carbon is due to organisms migrating and defecating at depth^49^. Because blooms originate on the west side of the Rise, where mixing of ST and SA waters begins and forms the frontal region, we hypothesize that the temporal evolution of salp blooms, accompanied by progressively increasing export flux, generally goes from west to east (Fig. 9).

Furthermore, based on both ^234^Th and DNA data, Non-salp SA (the last cycle) could also represent a location where salp blooms terminate, consistent with export in the whole region being affected by salp blooms in the summer. While studies comparing salp biomass to DNA read abundance have not been conducted, the read abundance patterns are informative because the biases are conserved across cycles and within each sampling method^50^. That *S. thompsoni* sequences are present throughout the water column and PIT, and that sediments show the highest amount of salp DNA in this area (3000 m depth) suggest repeated particle delivery and/or a very rapid transfer to depth, assuming a short life span for salp DNA on the seafloor. While it is hard to speculate on the DNA degradation time in sediments, and thus the time frame over which we can assume salp DNA is delivered to these locations, previous studies on microbial degradation of salp FP organic matter suggest degradation times of 0.02–0.04 d^−1^ (primarily at 5 °C) suggesting a pellet would degrade entirely over 24–40 days^51^. In addition, a study conducted on salp carcasses found rates of 1.65 d^−1^, suggesting most of a salp is degraded within a week at 15 °C^52^. Studies on the decomposition of extracellular DNA in marine sediments indicate they are higher than in the water column, which occurs on the order of a day^53,54^. Assuming the decomposition of salp DNA is similar to that of salp-derived organic matter (either FP or carcasses) or less, the DNA found in the cores (which was scraped from the surface) could have been deposited anytime from the day of collection to 40 days prior. Given the ^234^Th results for Non-salp SA, which suggest a high-flux event sometime in the prior 24 days, we believe it can be concluded that this DNA represents a recent bloom and not a much older event. The lack of salp DNA in sediments below Salp ST is somewhat surprising. However, studies have found that sedimentation near the top of the Chatham Rise is minimal, likely due to strong tidal and current forces acting on the crest of the rise^55^. In the region of Non-salp SA, other studies have also documented massive export of picoplankton embedded in large organic aggregates that were hypothesized to be produced by either small salps or appendicularians^56^.

The effect of salps on export pathways is evident not only in the magnitude of flux, but also in the composition of exported microplankton, as salp/non-salp explains significant levels of variance in NMDS analysis of PIT. While this relationship can be due to the link related to salp bloom affinity already established in the upper water column, our analysis of the similarity between communities in PIT and those living above indicate a significant role for salps in changing exported prey assemblages. The specific example of *G. oceanica* has additional implications for particulate inorganic carbon flux. This is the second most-ubiquitous coccolithophore after *Emiliana huxleyi*, and shares a similar size (6–10 µm) and ecological niche^57^, although its thermal preferences are restricted to waters above 13 °C^58,59^. In New Zealand waters, both *E. huxleyi* and *G. oceanica* can be major components of extensive coastal blooms^60^, and contribute to the export of inorganic carbon to depth^61^. The disproportionally high export of these prymnesiophytes during Salp ST could have been either through direct consumption and FP packaging, or by aggregating onto sinking particles, but regardless indicate salp effects on inorganic carbon flux when coccolithophores are present.

That Salp SA waters do not share the enhanced similarity between WC and PIT, compared to Salp ST and Salp SA-Sc, is somewhat surprising. Perhaps this is due to Non-salp SA sinking communities still showing the influence of the earlier export, as evidenced by high salp sequences within PIT and WC. Another plausible explanation is microbial degradation. While we expect salps, as generalist feeders^15,16,20^, to enhance the flux of smaller particles that otherwise do not sink, degradation and grazing by colonizing microbes alters both the quantity and composition of sinking particles^40,56^, processes that will obscur this effect in general. The relatively small enhancement of T_100_ in salp locations is consistent with significant reworking of FPs, which could also be due to production of loosely structured pellets^40^, that leads to slower sinking rates. These results suggest that the effect of salps on passive POC flux is generally experienced over this larger, regional scale.

The SalpPOOP study provides clear evidence for the role of salps in affecting phytoplankton growth dynamics and disproportionally enhancing export. The E_Z_ ratio in both SA/ST waters without salps is similar to traditional low-flux environments such as the North Pacific Subtropical Gyre^62^, while salp blooms shift the ecosystem to conditions comparable to high-flux regions such as the North Atlantic^63,64^ and the Southern Ocean Polar Frontal Zone during the austral diatom bloom in spring^3,65^. It should be noted that while this study only addresses salp-mediated export in the form of fecal pellets, export due to sinking carcasses and active diel vertical migration^66,67^ would suggest an even greater effect on the marine C-cycle when all pathways are considered. We note that investigations of changing Southern Ocean biogeochemistry often focus on impacts mediated by changing NPP. For instance, an export efficiency meta-analysis suggests that a doubling of NPP would increase carbon export by ~50% or less^68^. Our results highlight the importance of factors other than NPP as key players in Southern Ocean biogeochemistry. Zooplankton grazers, and their changing abundance and distribution patterns as a consequence of global warming, have the potential to not only alter marine food webs^7,69,70^, but also biogeochemistry^71–73^. If the increasing trend in salp abundance in the Southern Ocean persists^7^ at comparable rates, we can expect important changes in areas where salp blooms are recurrent: in the dynamics of phytoplankton bloom formation and termination, in the absorption and sequestration of carbon dioxide by the ocean, and in the composition of exported plankton affecting both organic and inorganic carbon flux to the deep ocean.

## Methods

### Oceanographic sampling

Sampling for SalpPOOP was carried out onboard the *R/V* Tangaroa from October 23 to November 21, 2018, in the vicinity of the Chatham Rise, east of New Zealand (Fig. 1). We used a Lagrangian experimental array to conduct in situ incubations, which consisted of surface floats for flotation and an iridium-enabled float for satellite tracking of the array. A plastic-coated wire connected the float to a 3 × 1-m holey-sock drogue centered at 15-m depth to ensure the array tracked the mixed layer. Mesh bags were placed to span the depth of the euphotic zone, adjusted for each cycle based on the conductivity–temperature–depth (CTD) fluorescence profile, and contained experimental rate measurement bottles at six depths (see Supplementary Table 6) within the euphotic zone deployed for 24 h. A surface-tethered, free-drifting particle interceptor trap (PIT) array was deployed in proximity to the in situ array, equipped with a surface Iridium beacon and light flasher, floats, 10 m-long bungies and holey-sock drogue at 15 m to ensure the trap arrays followed the same water parcel as the in situ array. A total of five water parcels were sampled, for periods ranging from 3 to 7.5 days (main text Table 1).

### Physical oceanography

Profiles of temperature, salinity, dissolved oxygen, and photosynthetically active radiation (PAR) were provided by a Seabird (SBE 911plus) CTD attached to a rosette frame with 24 10-L Niskin bottles for water collection. Samples for sensor calibration (salinity and dissolved oxygen) were taken throughout. Nutrient samples were taken at selected depths indicated in Supplementary Table 6. Filtered seawater used for nutrient analysis was obtained by gravity using a 0.2 μm Acrocap in-line capsule (Pall-Gelman) connected with acid-rinsed silicon tubing directly to the corresponding Niskin bottle. A sterile 50-mL falcon tube was rinsed with filtered seawater and filled to ~30 mL, sealed with parafilm, and stored at −80 °C until analysis. Nitrate (NO_3_^−^), ammonium (NH_4_^+^), dissolved reactive phosphorus (DRP) and silicate (DRSi) concentrations were measured using an Astoria Pacific API 300 microsegmented flow analyzer (Astoria‐Pacific, Clackamas, OR, United States) according to the colorimetric methods^74^. Analyses were done at NIWA Christchurch (New Zealand).

### Phytoplankton biomass, community composition, and physiological status

The phytoplankton community was sampled from CTD water bottle for chlorophyll *a* (total and size-fractionated at 20, 2, and 0.2 µm), and chemotaxonomic pigments using High-Performance Liquid Chromatography (HPLC), at depths detailed in Supplementary Table 6 and volumes detailed in Supplementary Table 7. For total chl *a* and HPLC, water from selected depths was filtered directly onto 25 mm GFFs and flash-frozen in liquid nitrogen. For size-fractionated chl *a*, 250–500 ml subsamples from 6 depths within the euphotic zone were size-fractionated on Nucleopore polycarbonate filters at 0.2, 2, and 20 μm, placed in cryovials and stored at −80 °C until analysis^75^. Pigment determinations are described below. The eukaryotic community composition was assessed using DNA metabarcoding of the 18 S rDNA. Below, we explain in more detail the methods associated with data in this study. Phytoplankton physiology was evaluated using a MiniFire fast repetition rate fluorometer (FRRF), using a sample volume of 5 ml (for depths and volumes, see Supplementary Tables 6 and 7). Fv/Fm and reoxidation of Q_a_, the first quinone acceptor of Photosystem II—PSII) were evaluated using the MiniFIRe, which provided indications of phytoplankton stress^76^.

#### ^14^C Net primary production (NPP)

Net primary production (NPP) was assessed using ^14^C assays^77^, with 24-h incubations that integrated respiration/production balance over the dark and light periods of the diel cycle. Seawater samples (1.3 L) were collected into an acid-rinsed polycarbonate bottle from pre-dawn CTD casts (~0200 h deployment) at six depths (Supplementary Table 6) spanning the euphotic zone. The bottles were then spiked with 0.1 mCi ^14^C-bicarbonate (DHI, Denmark or Perkin-Elmer, USA) before triplicate controls on ethanolamine were taken to quantify initial radioactivity at each depth incubation. After recovery, the entire content of the bottles were filtered onto 0.2-µm pore size 25-mm. acidified on land with 200 µL 0.5 N HCl, Hi Safe 3 liquid scintillation cocktail and disintegrations per minute determined using a scintillation counter^75^.

#### Phytoplankton growth and microzooplankton grazing

Rates of phytoplankton growth and microzooplankton grazing were assessed daily with the dilution technique^78^, following the two treatment approach^79^, at six depths within the euphotic zone. Seawater collected with the Niskin bottles attached to the CTD rosette at 0200 h was used to fill a pair of 2.2-L polycarbonate bottles (100%, B and C) while a third bottle (A) was filled with 25% whole seawater diluted with 0.2-µm filtered seawater from the same Niskin bottle. Nutrients (final concentrations in 2.2-L bottles; nitrate 0.18 μM, ammonium 4.16 μM, phosphate 15.08, silicate 44.2 μM, and vitamins) were added to bottles A and B in order to ensure dilutions assumptions are met^75^. Bottles were then incubated in situ at the same six depths of collection using a drifting array. Rates were calculated from changes in chl *a* and picophytoplankton abundance over 24 h assuming exponential growth. Photoacclimation effects were corrected from changes in cell chl *a* fluorescence estimated by flow cytometry during incubations as a proxy of cell chl *a* content^80^. FL3:FSC are the ratio of cell red fluorescence (680 nm) and forward side scatter measured by flow cytometry and used as a proxy of Chla:C ratio. Growth rates were corrected for photoacclimation using a photoacclimation correction factor (Phi) calculated using the average FL3:FSC in the initial and final picoeukaryotes and nanoeukaryotes populations weighted by their respective biomass. This combined Phi (Phi_peuk_nanoeuk) was then subtracted from the pigment-based instantaneous growth rate at each depth to estimate the in situ growth rate adjusted to photoacclimation rather than growth-related cell pigment changes during incubation experiments^81^.

### Pigments (water column, dilution, and zooplankton)

Chl *a* and phaeopigments (for water column biota, dilution experiments, and zooplankton gut contents) were determined using a Turner 10 AU fluorometer with chl *a* filter sets, using the acidification in vitro approach^82^. Seawater samples were filtered onto 25 mm GF/F filters and flash-frozen. Subsequently, filters were extracted in 7 mL of 90% acetone for 24 h at −20 °C, brought to room temperature and assayed. For zooplankton and salp gut pigment determinations, the organism or its guts were placed in a 15-mL Falcon tube containing 6 mL of 90% ice-cold acetone, sonicated, and extracted for 4–20 h. After this period, samples were centrifuged at 3000×*g* for 5 min, and chl *a* and phaeopigments were measured in the fluorometer. No correction for pigment destruction was applied^83^.

Samples for chemotaxonomic pigment determinations were shipped frozen to Instituto Español de Oceanografía, Centro Oceanográfico de Gijón, where they were extracted for HPLC analyses following established protocols^84^.

### 16S DNA metabarcoding

Bacterial community composition samples were collected from a minimum of six depths, filtering 0.5–1.5 L of seawater through a 0.2-μm 47 mm Millipore filter, flash-frozen in liquid nitrogen and stored at −80 °C until processing. DNA was extracted separately from each filter using a PowerSoil DNA Isolation Kit (Mo Bio, Carlsbad, CA, United States). The manufacturer’s protocol was modified to use a Geno/Grinder for 2 × 15 s instead of vortexing for 10 min. DNA concentration was measured using a Nanodrop Spectrophotometer and then a Qubit^TM^ DNA HS Assay Kit, both from Thermo Fisher Scientific. 16 S rRNA gene amplicon sequencing was carried out using the Earth Microbiome Project barcoded primer set and conditions^85^. All amplicons (independent replicates) were run on an Illumina HiSeq 151 bp x2 run. Amplicon sequence variants (ASVs) were then resolved at single-nucleotide resolution using the dada2 pipeline^86^. SILVA release 132 database^87^ was used to assign taxonomy. The phyloseq^88^ package in R (R Core Team, 2019) was used for sequence read counts, taxonomic assignments and associated metadata. Sequence reads were randomly rarified to an even depth of 14,900 reads per sample prior to analyses. Bray Curtis-based PERMANOVAs using the Adonis function in the vegan package^89^ were used to determine whether the communities were significantly different between water masses and salp blooms.

### 18S DNA metabarcoding of water column, PIT, and sediments

Samples from the water column were collected from six depths within the euphotic zone, filtering 1.4–2.4 L per depth. Samples from PITs were collected from four depths (see PIT section below), and typically contents of one full formalin-preserved sediment trap tube were filtered per sample. Sample collection consisted of filtering contents through 47 mm diameter polycarbonate filter for water column samples (0.2 μm pore size) and serially size-fractionated for PITs samples with 20-μm and 0.2-μm pore size filters (Poretics). Water from multiple depths were assayed using McLane pumps, assaying the same depths as the PIT, with typically 200 L of water collected onto polycarbonate (0.2)-μm filters). Sediment samples were collected using an 8 tube multicorer, once per cycle, and the material from the top (1 cm) of one tube was used for DNA extraction. The filter was flash-frozen in liquid nitrogen and stored at −80 °C until processing. DNA was extracted using Dneasy mini kit (Qiagen, Germany) – ‘Qiagen DNA easy Blood and tissue for PIT and water column, and PowerSoil (for sediments). PCR conditions followed a modified protocol^90^. Each 50 µL reaction included 25 pmol of each primer (V4F_Illumina—5’-CCAGCASCYGCGGTAATTCC-3’, V4Azig_Illumina—5’-ACTTTCGTTCTTGATYRATGA-3’), 1× KAPA HiFi HotStart ReadyMix (KAPA Biosystems), and 10–50 ng of template DNA, with a thermal profile of 95 °C for 3 min, followed by 10 cycles of 98 °C for 10 s, 44 °C for 20 s and 72 °C for 15 s, followed by 15 cycles of 98 °C for 10 s, 62 °C for 20 s and 72 °C for 15 s, with a final extension of 72 °C for 7 min. Products were visualized on an agarose gel and successful amplifications were submitted for further adapter ligation and indexing, prior to sequencing on an Illumina MiSeq. Bioinformatics was conducted using dada2^86^ and phyloseq^88^ packages, using the PR2 database version 4.12 (https://pr2-database.org/)^91^ for a taxonomic assignation. The DESeq2^42^ package was used to investigate changes in sequence abundance between groups of samples.

### ^238^Uranium-^234^thorium disequilibrium export flux estimates

Twice per cycle ^238^U:^234^Th disequilibrium measurements were taken using the standard small volume method^92,93^. Briefly, 4 L water samples were collected by CTD rosette. Sample pH was adjusted to <2 with concentrated HNO_3_, spiked with a yield tracer—10 dpm ^230^Th—and shaken vigorously. After 6–12 h, pH was adjusted to 8–9 with NH_3_OH. Co-precipitation with KmnO_4_ and MnCl_2_ (100 μL each at 7.5 g L^−1^ and 33 g L^−1^, respectively) was conducted 8–12 h prior to filtration on a QMA filter which was then dried at 45 °C and mounted in a Riso sample cup. Beta activity was measured with a Riso Ultra-Low Background Beta Multi-counter. After a final beta activity measurement >6 half-lives after collection, filters were digested in 8 M HNO_3_/10% H_2_O_2_, spiked with 5 dpm ^229^Th, and sonicated. Thorium was then selectively isolated by column chromatography (AG-X8), and isotope ratios (^229^Th:^230^Th) were determined by inductively coupled plasma mass spectroscopy (Thermo Element-2 at the National High Magnetic Field Laboratory, Florida, USA) and used to determine ^234Th^ yield. Total ^234Th^ activities were used in a 1D steady-state water column export model^33^ with corrections for turbulent mixing ($V=-\kappa \frac{{\partial }^{2}{A}_{{th}}}{\partial {z}^{2}}$). Non-steady state export fluxes were determined for cycle 2 from the rate of change in ^234Th^ inventory over the duration of a cycle^33^. Export fluxes were calculated at the depths corresponding to the PIT deployments, and from which the C:^234^Th ratios were determined (see above). Measurement uncertainties were propagated through all equations^33^.

### Salp and zooplankton abundance and biomass estimation

We conducted double oblique zooplankton net tows from 200 m water depth to the sea surface using a 0.7 m-diameter Bongo frame with paired 200-µm mesh nets, equipped with two General Oceanics flow meters to measure the filtered volume and a temperature-depth recorder. Tows were conducted at least twice daily (day and night), and average volumes filtered ranged between 150–500 m^−3^. Salp specimens were identified to species, using the keys in^94–96^, classified into oozooid or blastozooid stages, measured for total length, and corrected to oral to atrial length (OAL)^38^. A random subsample (10 specimens, when available) of each species/stage from each tow was taken for determination of chl *a* in salp guts for grazing estimates. For further biomass and chl *a* analyses, *Salpa thompsoni* lengths were divided into 5-mm bins (1–136 mm), abundance was calculated for each size bin, and biomass was calculated using length-frequency distributions^97^.

### Salp grazing

Salp specimens (typically ten of each species/stage if abundance allowed) from each tow had their guts excised, and chl *a* and phaeopigment gut content concentrations were measured. A power function was used to fit the size-specific Gpig (chl *a* + phaeo) contents for each tow, allowing the estimation of Gpig for each size bin per tow, and this was multiplied by the abundance in each size bin. Gut passage time (GPT) was calculated using a modified equation, based on a personal communication from Kremer^98^ where: GPT(h) = 2.607*ln(OAL, mm) – 2.6, and scaled by a Q_10_ = 2. Grazing was estimated as: G (h^−1^) = Gpig /GPT. Daily salp grazing rates were obtained by assuming 14 h of day and 10 h of night.

### Fecal pellet production rates

We calculated the FP production rates based on our in situ data, as well as using the relationship derived for Antarctic waters for comparison^40^. Fprod_Gpig_ is based on the grazing estimation detailed above, multiplied by an egestion efficiency (EE) of 0.36, based on assimilation efficiencies (1-EE) of 0.64 (range 0.44–0.73) derived for *S. thompsoni* in the Lazarev sea^39^, which is within the range of other studies (range AE = 0.55–0.75)^99,100^. Error estimates are standard errors from all the tows conducted in a cycle. For Fprod_Iversen_ we use the published FP production rate and carbon per pellet conversions as a function of salp size (size, mm)^40^: Fprod (pellets h^−1^) = 0.5388 * e^(−0.0212*size*)*^, and OrgCpellet (µg C pellet^−1^) = 0.055*size^2.0665^. A Q_10_ = 2 was applied to account for the difference in temperature between the two studies. For day/night production rates we used the day/night abundance averages, and multiplied by the same factors as above (14/10) hours for day and night, respectively. Error for Fprod_Iversen_ was propagated from the abundance estimates based on all tows within each cycle.

### Passive export flux—particle interceptor sediment traps (PIT)

Surface-tethered, free-drifting cylindrical PITs (inner diameter 7 cm; 8:1 height: diameter aspect ratio) were deployed at the start of each experimental cycle^101,102^. Traps were topped by a baffle constructed from smaller 1.3 cm-diameter tubes with a similar 8:1 aspect ratio. Cross-frames, holding 12 baffled PITs, were typically deployed at ~30 m below the base of the mixed layer (as estimated by CTD profiles (T, S, density and fluorescence) and at 100 m, 300 m, and 500 m below the sea surface. PITs were filled with SupraPak 0.2-µm cartridge-filtered seawater and then backfilled to a height of one cylinder diameter (~7–8 cm) with hypersaline brine solution (filtered seawater + 50 g L^−1^ NaCl) either with or without buffered formalin (0.4% formaldehyde final concentration) depending on intended analysis. Upon recovery, the overlying seawater was gently siphoned off before the samples were filtered through 200-µm mesh. The mesh filters were then examined under a dissecting microscope (×20 magnification) and zooplankton “swimmers” were removed manually prior to photographing the >200 µm fraction. Both size fractions were then recombined, and samples were either filtered on pre-combusted GF/F for particulate organic carbon and nitrogen (POC, PON), uncombusted GF/F for chl-*a*, and QMA or membrane filters for C:^234^Th_p_ ratios or DNA metabarcoding (see below). Additional samples were collected for microscopy. POC and PON were determined on acidified (fumed, HCl) samples run on an isotope ratio mass spectrometer at the UC Davis Stable Isotope Facility (USA). To calculate flux at all depths (z, m) we assumed a power law distribution of the form Flux (z) = F_0_*(*z*/z_0_)^−b^, with F_0_ representing flux below the euphotic zone. Results are relatively insensitive to the shape of the functional form used (i.e., power law or exponential).

### Salp fecal pellet contribution to PIT fluxes

The >200-μm mesh filters for each PIT tube (used for removing zooplankton “swimmers”) were imaged using a Canon 5D Mark II camera with attached 100 mm F/2.8 macro lens mounted in a downward-facing macrophotography rig. Images were manually analyzed using Image J to determine morphometric measurements for each large salp fecal pellet (FP). Morphometric measurements were then used to estimate FP volume and carbon content.

### Fecal pellet carbon:volume and sinking rates

Salp FPs (*n* = 98) were collected from live incubations of salps to: (i) measure carbon:volume FP relationships. FPs had their length and width measured, and were measured for carbon and nitrogen to create a carbon: volume relationship, which was applied to the pellets imaged in the PITs to estimate the salp FP contribution to export. Carbon content of each FP was determined from a log–log relationship between pellet volume and pellet carbon mass ($C={10}^{0.634\times {{{\log }}}_{10}\left(V\right)+1.43}$, where C is carbon mass in units of μg carbon and V is volume in mm^3^).

### Multicorer

At each cycle, an Ocean Instruments MC-800 multicorer was deployed to the ocean bottom to obtain core samples. Typically, 6 core tubes (~10 cm diameter) were deployed once per cycle in order to complete the required sampling. Prior to sediment sampling, seawater was aspirated off each core to a height just above (~1 cm) the sediment surface. Samples for DNA metabarcoding were taken from 1 core tube by conducting a surface scrape that removed 25 ml of sediments, placing in a 50-ml falcon tube, and freezing in a −80 °C freezer.

### Statistical analyses

Bioinformatic analyses were carried out in R. Plots of rates, and standing stocks were carried out in MATLAB, as well as Model I regressions.

### Reporting summary

Further information on research design is available in the Nature Portfolio Reporting Summary linked to this article.

## Supplementary information

Supplementary Information

Reporting Summary

## Data Availability

Export flux data and ^238^U:^234Th^ are deposited in BCO-DMO (project 754878). Salp abundance, salp gut pigments, chl *a*, size-fractionated chl *a*, phytoplankton physiology, integrated HPLC, and phytoplankton growth and grazing has been deposited in PANGAEA. https://www.bco-dmo.org/project/754878; 10.26008/1912/bco-dmo.813759.1; 10.26008/1912/bco-dmo.813859.1; 10.26008/1912/bco-dmo.813828.1; 10.1594/PANGAEA.928084; 10.1594/PANGAEA.928086; 10.1594/PANGAEA.928087; 10.1594/PANGAEA.928088; 10.1594/PANGAEA.928089; 10.1594/PANGAEA.928092; 10.1594/PANGAEA.928096. All bioinformatics data have been deposited in NCBI (16S data—PRJNA670059 and 18S (CTD and Traps)—PRJNA670061). The remaining datasets presented in the manuscript are available in the tables.
