# Supplementary material for: Salp blooms drive strong increases in passive carbon export in the Southern Ocean
Source: Nat Commun. 2023 Feb 2;14:425. doi: 10.1038/s41467-022-35204-6 (PMC9894854; doi:10.1038/s41467-022-35204-6)
Supplement: Supplementary file 1 — Supplementary Information [file 41467_2022_35204_MOESM1_ESM.pdf]

Supplementary Information

Salp blooms drive strong increases in passive carbon export in the Southern Ocean

Moira Décima, Michael R. Stukel, Scott D. Nodder, Andrés Gutiérrez-Rodríguez, Karen E. Selph, Adriana Lopes dos Santos, Karl Safi, Thomas B. Kelly, Fenella Deans, Sergio E. Morales, Federico Baltar, Mikel Latasa, Maxim Y. Gorbunov, and Matt Pinkerton

Correspondence to: mdecima@ucsd.edu

Supplementary Figures

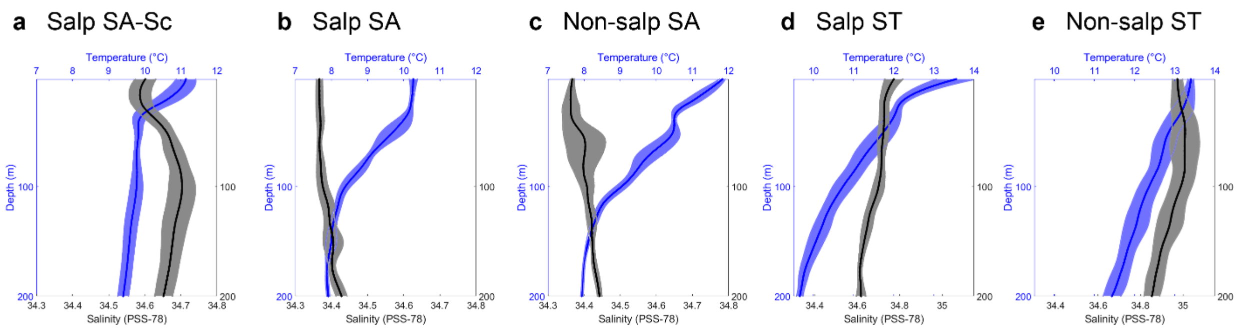

Supplementary Fig. 1. Temperature and salinity profiles (± std) for all five experimental locations. **a-c** SA locations, **d-e** ST locations. Note difference in x-axes in SA compared to ST waters.

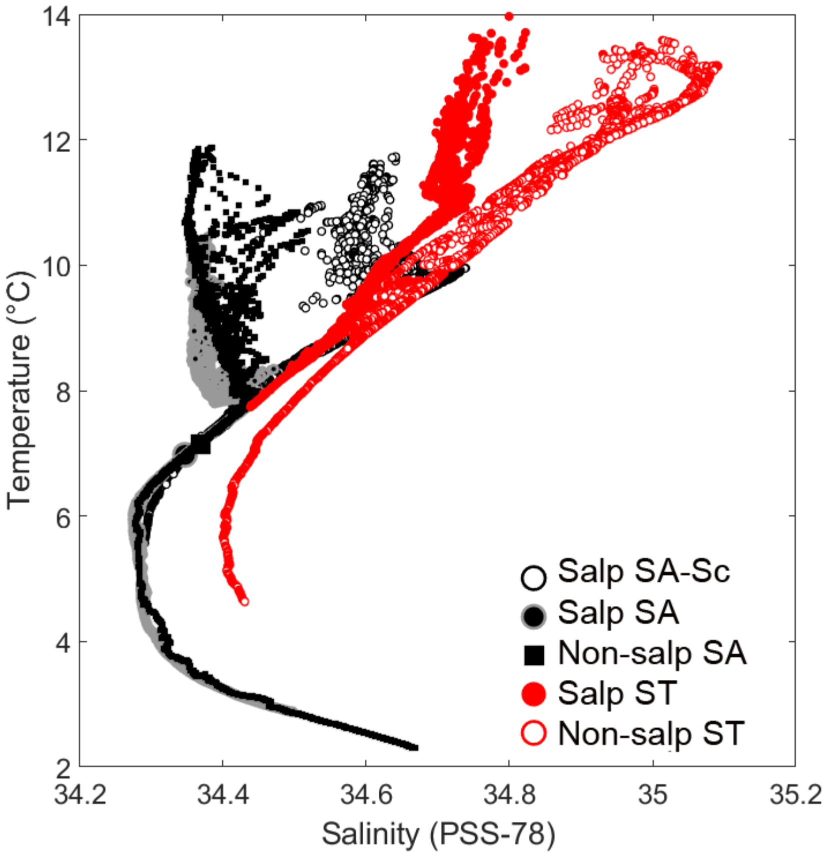

Supplementary Fig. 2. Temperature- Salinity for SalpPOOP cycles. Black and grey areas represent SA conditions, and red are ST areas.

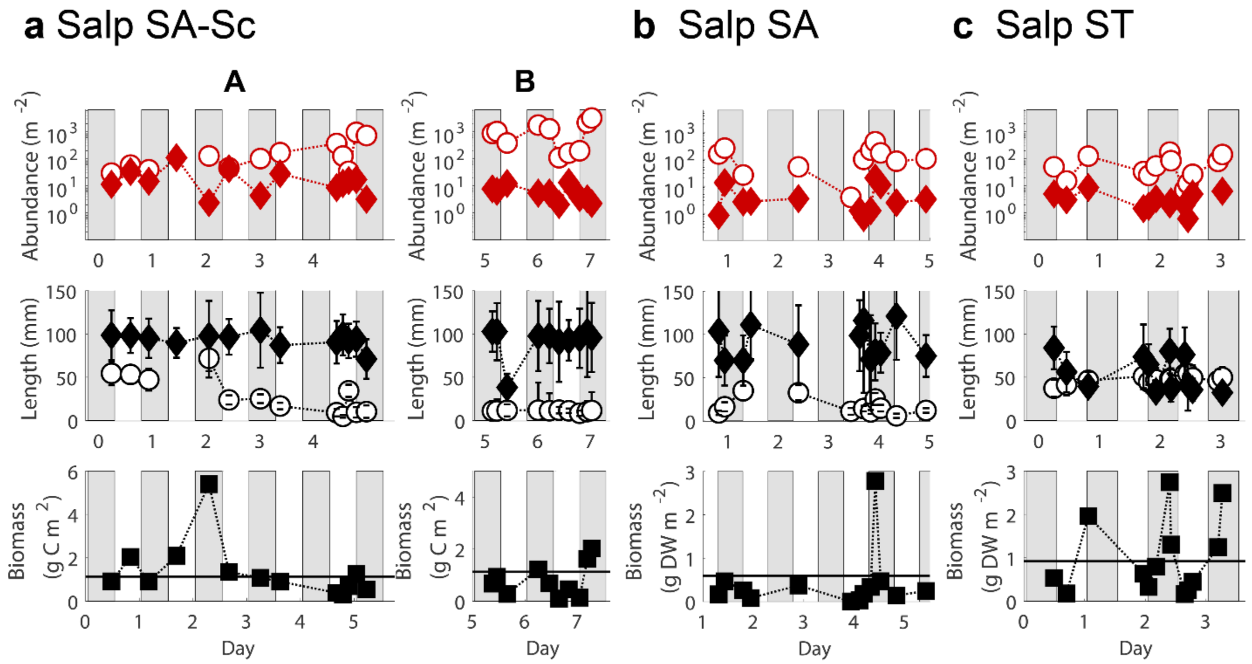

Supplementary Figure 3. Abundances (salps m^{-2}), mean (± weighed standard deviation) OAL, and biomass *S. thompsoni*, oozooids in diamonds, blastozooids in open circles, total biomass in black squares (line through indicates average over the cycle). **a** Salp SA-Sc, divided into Period A (first 5 days) and Period B (subsequent 2.5 days), **b** Salp SA, and **c** Salp ST. Grey bars indicate night times, white bars indicate day times.

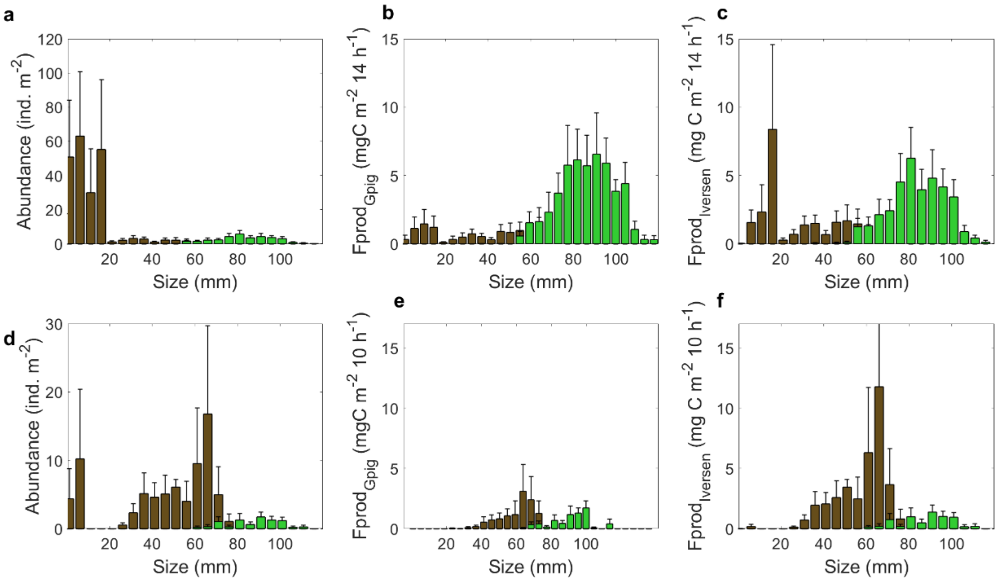

Supplementary Figure 4. Size-binned abundance and fecal pellet production estimates for experimental cycle Salp Subantartctic Southland Current (SA-Sc) A. **a** Daytime abundance (ind m^{-2}), **b** daytime fecal pellet production using Fprod$_{Gpig}$ (mg C m^{-2} 14 h^{-1}), **c** daytime fecal pellet production using Fprod$_{Inversen}$ (mg C m^{-2} 14 h^{-1}), **d** nighttime abundance (ind m^{-2}), **e** nighttime fecal pellet production using Fprod$_{Gpig}$ (mg C m^{-2} 10 h^{-1}), **f** nighttime fecal pellet production using Fprod$_{Inversen}$ (mg C m^{-2} 10 h^{-1}). Brown indicates values for blastozooids and green indicates values for oozooids.

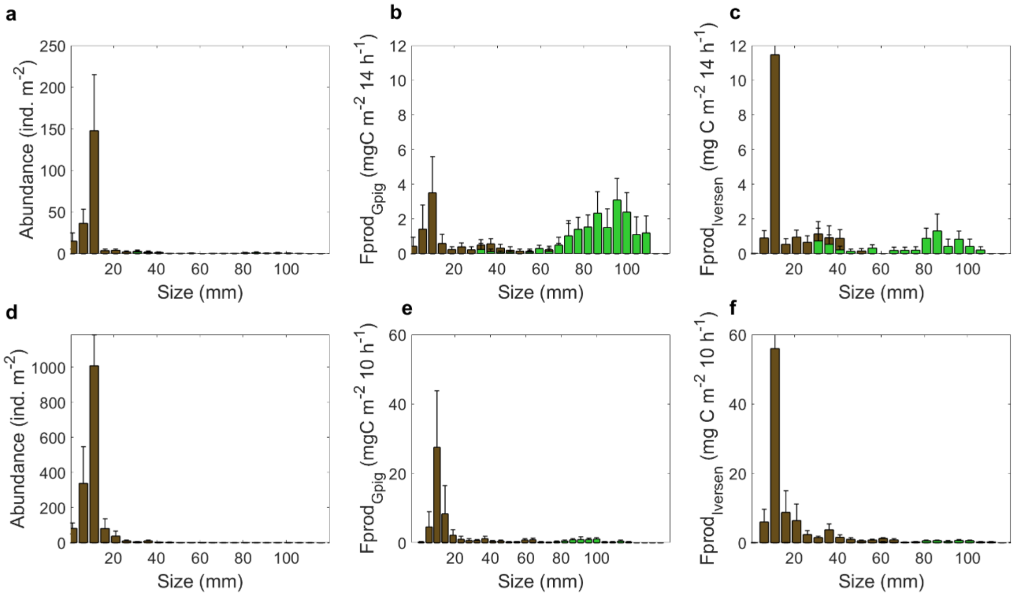

Supplementary Figure 5. Size-binned abundance and fecal pellet production estimates for experimental cycle Salp Subantartctic Southland Current (SA-Sc) B. **a** Daytime abundance (ind m^{-2}), **b** daytime fecal pellet production using Fprod$_{Gpig}$ (mg C m^{-2} 14 h^{-1}), **c** daytime fecal pellet production using Fprod$_{Inversen}$ (mg C m^{-2} 14 h^{-1}), **d** nighttime abundance (ind m^{-2}), **e** nighttime fecal pellet production using Fprod$_{Gpig}$ (mg C m^{-2} 10 h^{-1}), **f** nighttime fecal pellet production using Fprod$_{Inversen}$ (mg C m^{-2} 10 h^{-1}). Brown indicates values for blastozooids and green indicates values for oozooids.

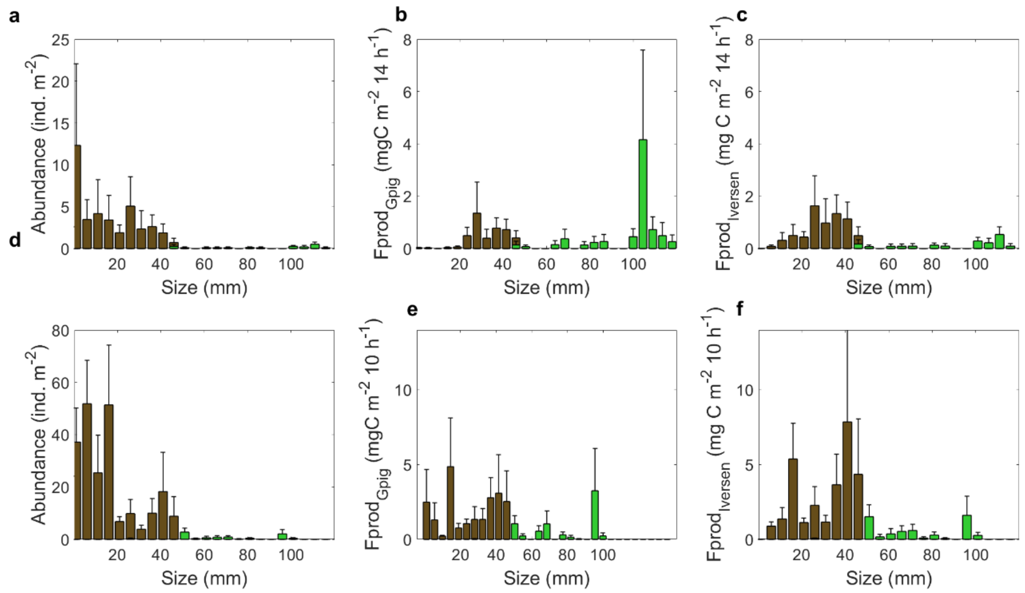

Supplementary Figure 6. Size-binned abundance and fecal pellet production estimates for experimental cycle Salp Subantarctic (SA). **a** Daytime abundance (ind m^{-2}), **b** daytime fecal pellet production using $Fprod_{Gpig}$ (mg C m^{-2} 14 h^{-1}), **c** daytime fecal pellet production using $Fprod_{Inversen}$ (mg C m^{-2} 14 h^{-1}), **d** nighttime abundance (ind m^{-2}), **e** nighttime fecal pellet production using $Fprod_{Gpig}$ (mg C m^{-2} 10 h^{-1}), **f** nighttime fecal pellet production using $Fprod_{Inversen}$ (mg C m^{-2} 10 h^{-1}). Brown indicates values for blastozooids and green indicates values for oozooids.

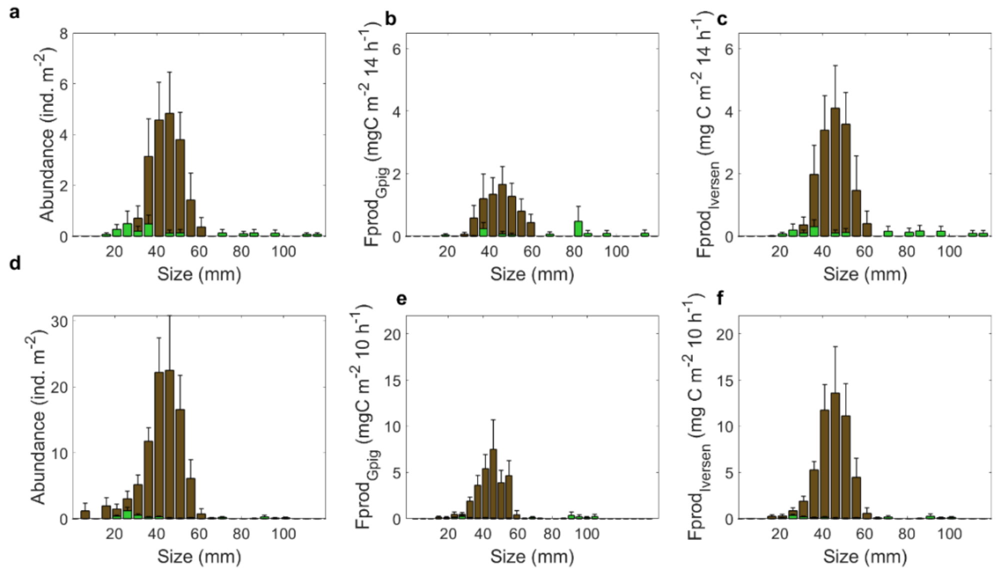

Supplementary Figure 7. Size-binned abundance and fecal pellet production estimates for experimental cycle Salp Subtropical (ST). **a** Daytime abundance (ind m^{-2}), **b** daytime fecal pellet production using $Fprod_{Gpig}$ (mg C m^{-2} 14 h^{-1}), **c** daytime fecal pellet production using $Fprod_{Inversen}$ (mg C m^{-2} 14 h^{-1}), **d** nighttime abundance (ind m^{-2}), **e** nighttime fecal pellet production using $Fprod_{Gpig}$ (mg C m^{-2} 10 h^{-1}), **f** nighttime fecal pellet production using $Fprod_{Inversen}$ (mg C m^{-2} 10 h^{-1}). Brown indicates values for blastozooids and green indicates values for oozooids.

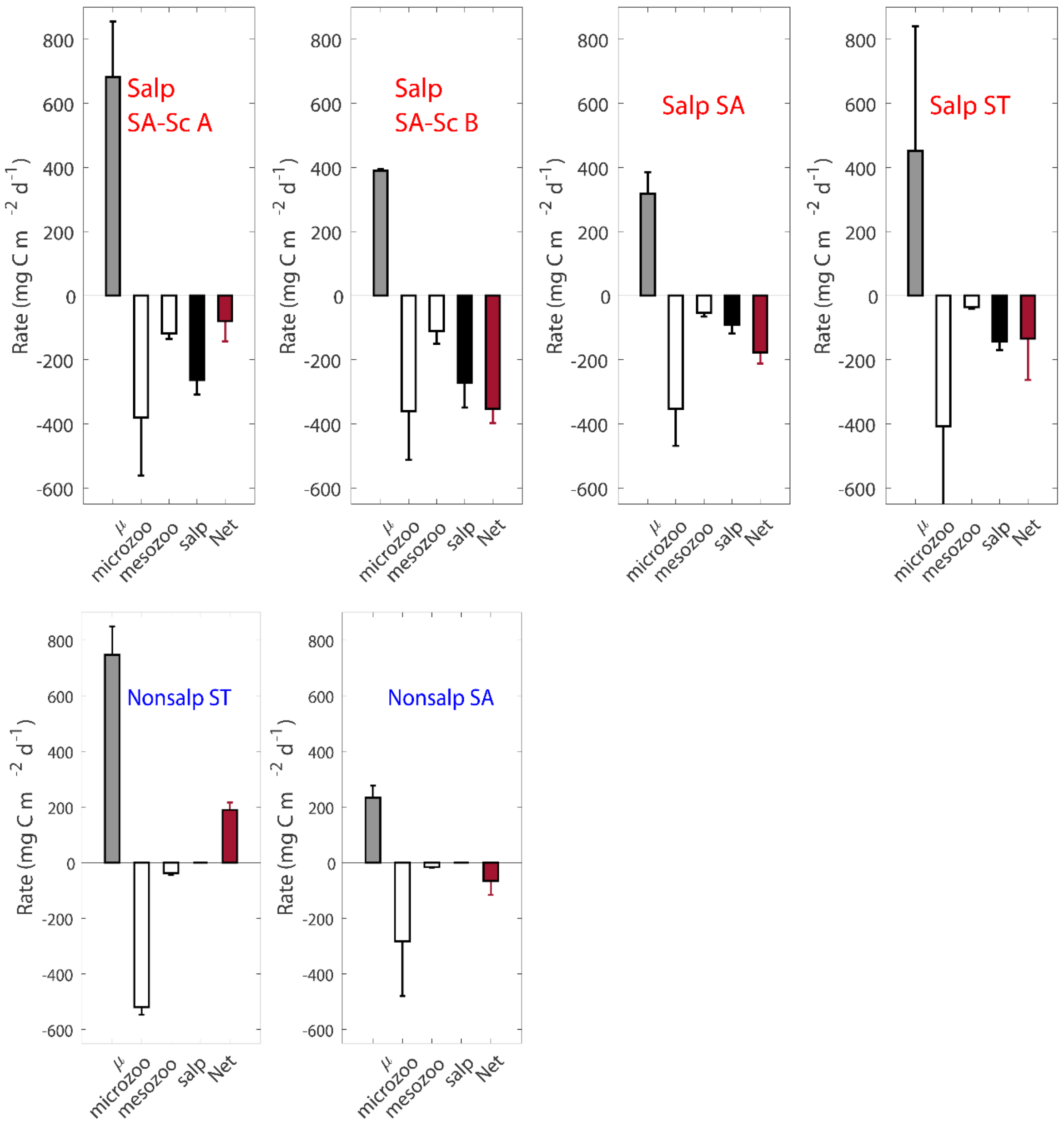

Supplemental Figure 8. Growth and grazing balances of salp cycles. Rates are ordered as: ^{14}C PP (μ), grazing by microzooplankton (microzoo), grazing by mesozooplankton (non-salp, mesozoo), grazing by salps (in black, salp), and the net rate of change (red, Net) which is the sum of all the rates. Error bars are propagated SE.

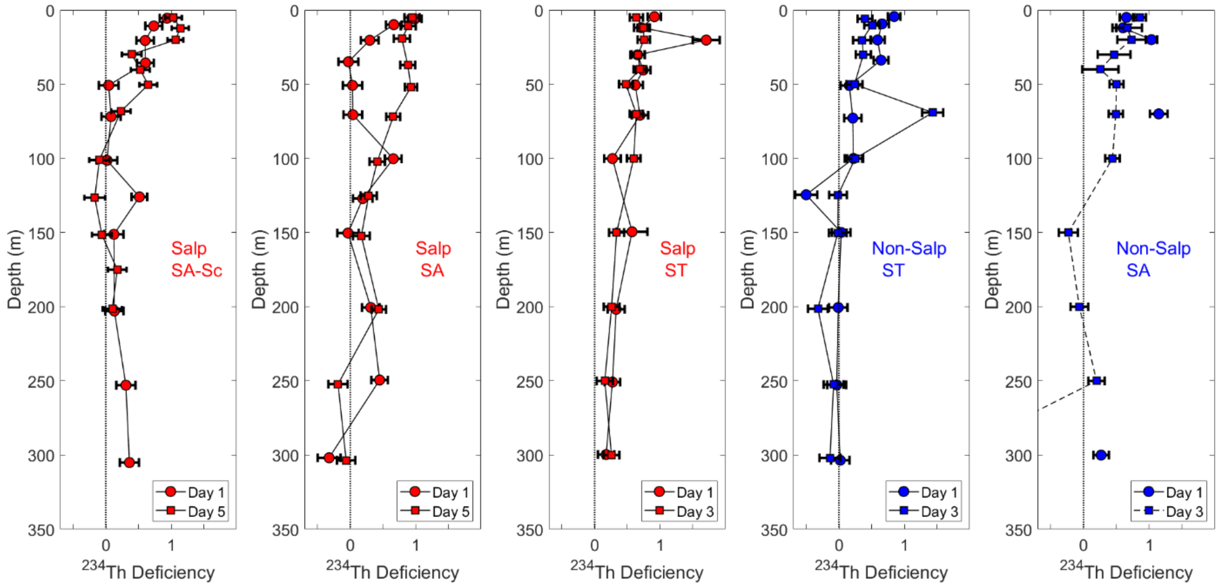

Suplemental Figure 9. ^{238}U:^{234}Th disequilibrium. Profiles from each cycle, two casts per cycle. Error bars are propagated from uncertainty in dpm[1]. Dotted line indicates the zero value, and the depth at which the profile crosses this line is indicative of the remineralization depth.

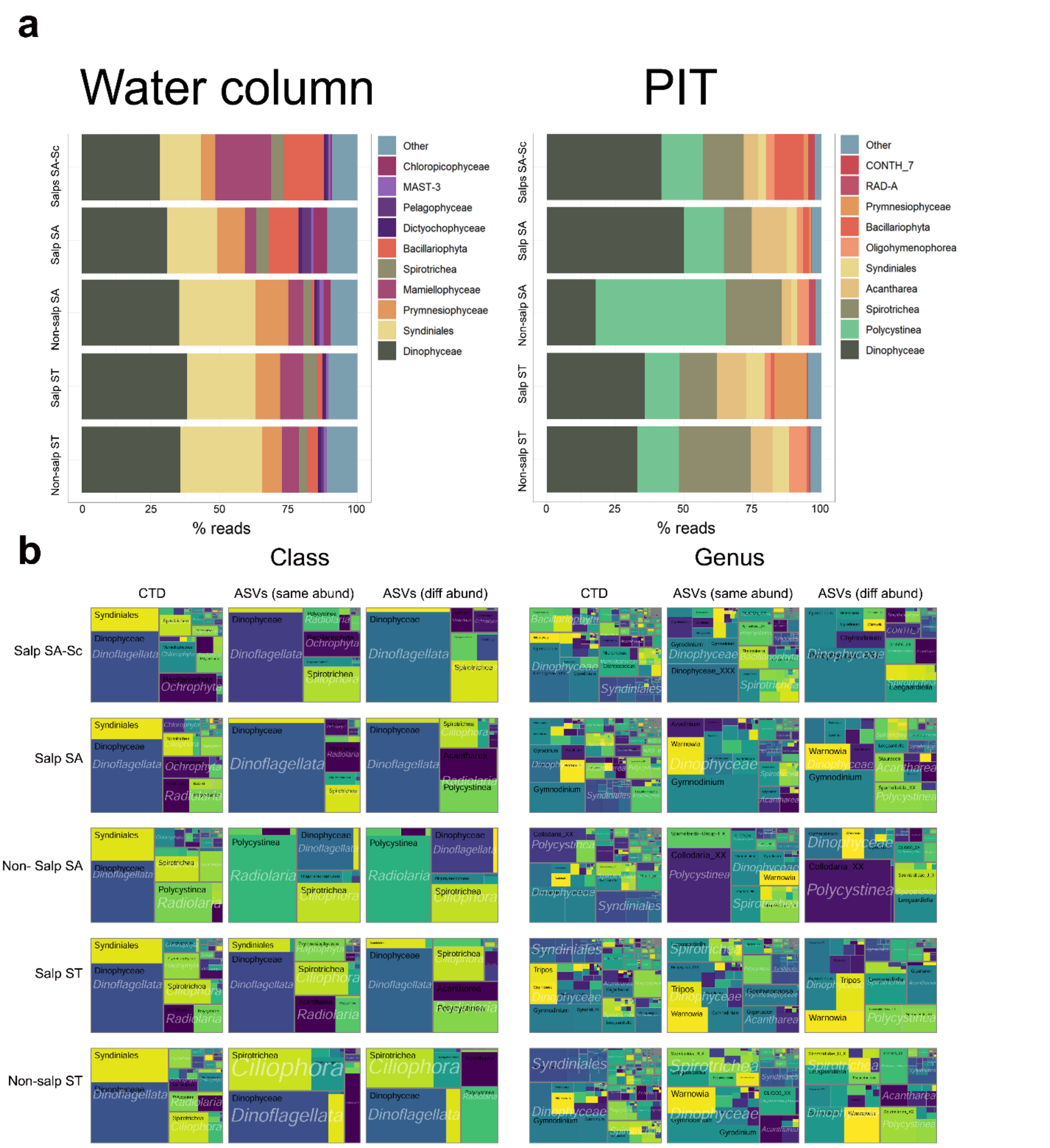

Supplementary Fig. 10. 18s ASVs is water column and sediment traps (PIT). **a** Percent DNA meta-barcoding read contributions of the main 10 plankton groups in water column and PIT samples. Water column includes six depths spanning the euphotic zone, while PIT sequences are from formalin-fixed sediment trap samples at four depths (see text for details). **b** ASV community composition (all, not restricted to phytoplankton) using class (left grouping) and genus (right grouping) classification. First column of each grouping corresponds to the ASVs from the water column, middle column includes ASVs that are common to both water column and PIT samples, and left column are ASVs that are statistically more abundant in PIT compared to the water column.

Supplementary Tables

Pigment	Abbreviation	Taxonomic significance
19' Butanoyloxyfucoxanthin	19But	Pelagophytes, Prymnesiophytes
19' Hexanoyloxyfucoxanthin	19Hex	Prymnesiophytes
Alloxanthin	Allox	Cryptophytes
Chl *b*	Chl b	Chlorophytes, Prasinophytes
Divinyl chl *a*	DVChla	Prochlorophytes
Fucoxanthin	Fuco	**Diatoms**, Prymnesiophytes and Pelagophytess
Peridinin	Per	Dinoflagellates
Prasinoxanthin	Pra	Prasinophytes
Zeaxanthin	Zea	Cyanobacteria

Supplementary Table 1. HPLC pigments and main chemotaxonomic phytoplankton group affiliations[2].

Cruise/Location	Sampling	Euphotic zone depth (m)	Flux at Ez (mg C m^{-2} d^{-1})	EZ	T_{100}
SalpPOOP	Salp SA-Sc A	70	77	0.11	0.65
SalpPOOP	Salp SA-Sc B	70	82.4	0.21	0.71
SalpPOOP	Salp SA	70	132.3	0.415	0.66
SalpPOOP	Non-salp ST	70	33.7	0.045	0.7
SalpPOOP	Salp ST	70	209.6	0.46	0.57
SalpPOOP	Non-salp S&A	110	25.5	0.11	0.52
K2	D1	50	133	0.25	40
K2	D1	50	39	0.11	54
ALOHA	-	125	15	0.07	67
NABE	-	50	493	0.45	100
EQPAC	-	120	26	0.02	61
KIWI	7	30	284	0.29	32
KIWI	8	60	488	0.34	80
OSP	May	60	31	0.03	31
OSP	August	40	97	0.14	32

Supplementary Table 2. Values plotted in Fig. 5e. Note data from cruises that are not SalpPOOP are from Buesseler and Boyd (2009)[3].

$Fprod_{Gpig}$	Oozooid	Blastozooid	All	Export @200m	Salp pellets @200	% Direct sinking	% Blastozooid contribution
Salp SA-Sc A	56.5 ± 21.2	24.5 ± 12.0	81.0 ± 24.3	50.9 ± 13.3	24.4 ± 16.1	30.1 ± 21.8	30.2 ± 17.3
Salp SA-Sc B	17.9 ± 8.6	77.7 ± 46.4	95.6 ± 47.2	59.1 ± 21.5	17.8 ± 7.8	18.6 ± 12.3	81.2 ± 63.0
Salp SA	14.3 ± 8.3	26.2 ± 15.3	40.5 ± 17.4	86.4 ± 38.1	26.8 ± 10.9	66.1 ± 39.2	64.8 ± 46.8
Salp ST	3.1 ± 1.9	36.1 ± 10.8	39.2 ± 11.0	101.8 ± 38.2	44.1 ± 12.9	112.6 ± 45.6	92.1 ± 37.7

$Fprod_{Iversen}$	Oozooid	Blastozooid	All	Export @200m	Salp pellets @200	% Direct sinking	% Blastozooid contribution
Salp SA-Sc A	43.2 ± 15.4	66.0 ± 32.8	109.3 ± 36.3	50.9 ± 13.3	24.4 ± 16.1	22.3 ± 16.5	60.5 ± 36.1
Salp SA-Sc B	12.9 ± 6.1	130.3 ± 51.5	143.1 ± 51.8	59.1 ± 21.5	17.8 ± 7.8	12.4 ± 7.1	91.0 ± 48.8
Salp SA	7.6 ± 4.4	39.8 ± 20.8	47.4 ± 21.2	86.4 ± 38.1	26.8 ± 10.9	56.5 ± 34.2	83.9 ± 57.7
Salp ST	3.9 ± 2.3	73.4 ± 19.5	77.3 ± 19.6	101.8 ± 38.2	44.1 ± 12.9	57.0 ± 22.1	94.9 ± 34.9

Supplementary Table 3. FP production calculated using grazing estimates from SalpPOOP ($Fprod_{Gpig}$) and the Iversen et al. (2017)[4] relationship ($Fprod_{Iversen}$). Values are in mg C m^{-2} d^{-1}. % Direct sinking indicates the percent of FP production that is collected as Salp pellets @200m. % Blastozooid contribution refers to the contribution to the total FP production. FP production values are mean ± SE.

	Plankton stocks and rates	Export 70m		Export 100m		Export 300m		Export 500m		Average	
		R	*p*-value	R	*p*-value	R	*p*-	R	*p*-value	R	*p*-value
Zooplankton	Zooplankton grazing	0.14	0.79	0.37	0.47	0.14	0.80	0.58	0.31	0.18	0.73
	Log zooplankton biomass	-0.10	0.85	0.17	0.75	-0.05	0.92	0.18	0.78	-0.03	0.95
Salps	Salp grazing	0.26	0.62	0.51	0.30	0.22	0.67	0.76	0.13	0.31	0.54
	Log salp abundance	0.42	0.40	0.64	0.17	0.42	0.41	0.72	0.17	0.47	0.34
	**Log salp biomass**	0.66	0.15	**0.63**	**0.06**	0.65	0.16	0.77	0.13	0.69	0.13
Chl *a*	Log Surface chl *a*	0.11	0.84	0.16	0.76	0.21	0.69	-0.17	0.79	0.14	0.79
	Log Areal chl *a*	0.01	0.99	0.02	0.97	0.13	0.81	-0.31	0.61	0.04	0.95
Nutrients	Areal nitrate	-0.34	0.51	-0.36	0.49	-0.44	0.39	0.05	0.93	-0.37	0.47
	Areal silicate	-0.47	0.34	-0.40	0.43	-0.54	0.27	0.05	0.93	-0.49	0.33
Phytoplankton rates and physiology	Average Fv/Fm	0.17	0.79	0.07	0.92	0.30	0.62	-0.47	0.53	0.17	0.78
	Average PSII reaction center	-0.28	0.65	-0.25	0.68	-0.40	0.51	0.19	0.81	-0.30	0.63
	Surface NPP	-0.24	0.64	-0.22	0.68	-0.16	0.77	-0.46	0.44	-0.23	0.67
	Areal NPP	-0.28	0.60	-0.23	0.66	-0.18	0.73	-0.44	0.46	-0.26	0.62
	Biomass accumulation	-0.11	0.86	-0.08	0.89	-0.02	0.98	-0.23	0.77	-0.10	0.87
	Chl *a* accumulation	-0.38	0.45	-0.36	0.48	-0.30	0.57	-0.46	0.43	-0.38	0.46
	Phytoplankton growth	-0.17	0.75	-0.05	0.93	-0.10	0.85	-0.09	0.88	-0.14	0.79
	Microzooplankton grazing	0.06	0.90	0.29	0.57	0.11	0.84	0.27	0.65	0.12	0.83
18S DNA dominant phytoplankton composition	Mamiellophyceae	-0.02	0.98	-0.04	0.95	0.05	0.94	-0.10	0.90	-0.02	0.97
	Dinophyceae	0.22	0.72	-0.03	0.96	0.28	0.65	-0.61	0.39	0.19	0.76
	Syndiniales	-0.29	0.64	-0.46	0.43	-0.23	0.71	-0.69	0.31	-0.31	0.61
	Prymnesiophyceae	0.01	0.99	-0.08	0.89	-0.08	0.90	0.06	0.94	-0.02	0.97
	Bacillariophyta	0.01	0.98	0.27	0.65	-0.03	0.96	0.64	0.36	0.05	0.94
HPLC phytoplankton composition	19' Butanoyloxyfucoxanthin	-0.14	0.82	-0.08	0.90	-0.26	0.67	0.30	0.70	-0.15	0.82
	19' Hexanoyloxyfucoxanthin	-0.14	0.82	-0.13	0.84	-0.24	0.70	0.13	0.87	-0.15	0.81
	Alloxanthin	-0.31	0.61	-0.39	0.51	-0.20	0.75	-0.70	0.30	-0.31	0.61
	Chl *b*	0.72	0.17	0.44	0.46	0.74	0.15	-0.10	0.90	0.68	0.21
	Divinyl chl *a*	-0.29	0.64	-0.25	0.68	-0.41	0.50	0.20	0.80	-0.30	0.62
	Fucoxanthin	0.13	0.84	0.25	0.69	0.14	0.82	0.33	0.67	0.14	0.82
	Peridinin	-0.19	0.76	0.14	0.82	-0.25	0.69	0.47	0.53	-0.13	0.83
	Prasinoxanthin	0.53	0.36	0.27	0.66	0.65	0.24	-0.71	0.29	0.51	0.38
	Zeaxanthin	-0.43	0.47	-0.47	0.42	-0.51	0.38	-0.13	0.87	-0.45	0.44

Supplementary Table 4. Regression results of plankton stocks and rates as explanatory variables of POC export fluxes at four depths, and average export flux for all five experimental cycles. Note that export at 500m does not include Salp ST because export was not measured that deep at that location. The only correlation that had $p<0.1$ is shown in bold; no variables were significant at $p<0.05$.

Supplementary Table 5. Results from DESeq2 analysis. ASVs in common between both types of samples, significant or not significantly different in abundance ($p < 0.01$). $\chi2$ results for the 2 SA comparisons and ST comparison. Significant differences in bold ($p < 0.01$).

	# ASVs not sig. different	# ASVs sig different	Comparison	Cycles	$\chi2$	*p* -value
**Salp SA-Sc**	553	142				
Salp SA	496	237	**SA**	**Salp SASc vs Non-salp SA**	**31.5**	**1.9 x 10^{-8}**
Non-salp SA	387	275	SA	Salp SA vs Non-salp SA	1	0.3
**Salp ST**	353	154	**ST**	**Salp ST vs Non-salp ST**	**14.9**	**0.0001**
Non-salp ST	319	173	Control	Non-salp SA vs Non-salp ST	4.4	0.03

Supplementary Table 6. Depths of water column sampling for each assay/instrument. Organized by cycle order.

Cycle order	Cycle name	Chl *a*/ size-fractionated chl a	^{14}C PP/ Dilution - based growth & grazing	Nutrients	HPLC	Phytoplankton physiology	PIT/MCLane	^{238}U: ^{234}Th
		Depths (m)	Depths (m)	Depths (m)	Depths (m)	Depths (m)	Depths (m)	Depths (m)
1	Salp SA-Sc	5, 12, 20, 30, 40, 50, 70, 100	5, 12, 20, 30, 40, 50	5, 10, 25, 40, 50, 70, 100	5, 12, 20, 30, 40, 50, 70, 100	5, 12, 20, 30, 45, 50	70, 100, 300, 500	5,12,20, 30, 40, 50, 70, 100, 150, 200, 250, 300
2	Salp SA	5, 12, 20, 30, 40, 50, 70, 100	5, 12, 20, 30, 40, 60	10, 25, 40, 50, 60, 70, 80, 100	5, 12, 20, 30, 40, 60, 80, 100	5, 12, 20, 30, 45, 60	70, 100, 300, 500	5,12,20, 30, 40, 50, 70, 100, 150, 200, 250, 300
3	Non-salp ST	5, 12, 20, 30, 40, 60, 80, 100	5, 12, 20, 30, 40, 50	10, 25, 35, 40, 50, 60, 70 100	5, 12, 20, 30, 40, 50, 70, 100	5, 12, 20, 25, 30, 35, 45, 50	70, 100, 300, 500	5,12,20, 30, 40, 50, 70, 100, 150, 200, 250, 300
4	Salp St	5, 12, 20, 25, 30, 40, 70, 100	5, 12, 20, 30, 40, 50	5, 12, 20, 30, 40, 50, 70, 100	5, 12, 20, 30, 40, 50, 70, 100	5, 12, 20, 30, 40, 50, 70	70, 100, 200, 300	5,12,20, 30, 40, 50, 70, 100, 150, 200, 250, 300
5	Non-salp SA	5, 12, 20, 30, 40, 50, 70, 100	5, 12, 30, 50, 60, 70, 90	5, 12, 24, 45, 70, 90, 100, 160	5, 12, 30, 50, 60, 70, 100, 120	5, 12, 25, 30, 45, 50, 60, 70, 90	110, 140, 340, 540	5,12,20, 30, 40, 50, 70, 100, 150, 200, 250, 300

Supplementary Table 7. Volumes filtered for each assay. Note that for some measurements, such as McLane pumps or DNA estimates, volumes were more variable due to filter loading or instrument limitations (slower filtration rates on McLane pumps), we have thus listed the general range of the volume filtered for that assay.

Analysis	Volume sampled (L)
Size-fractionated Chl a	0.2-0.5
Chl a	0.28
HPLC	1-2
Nutrients	0.03
^{14}C PP	1.3
Phytoplankton physiology	0.005
DNA - water column	1-2
DNA- PIT	1
DNA - McLane pump	150-450
^{238}U: ^{234}Th	4

References
1 Savoye, N. *et al.* ^{234}Th sorption and export models in the water column: A review. *Mar. Chem.* **100**, 234-249, doi:https://doi.org/10.1016/j.marchem.2005.10.014 (2006).
2 Wright, S. W. & Jeffrey, S. W. in *Marine Organic Matter: Biomarkers, Isotopes and DNA* (ed J.K. Volkman) 71-104 (Heidelberg, 2006).
3 Buesseler, K. O. & Boyd, P. W. Shedding light on processes that control particle export and flux attenuation in the twilight zone of the open ocean. *Limnol. Oceanogr.* **54**, 1210-1232, doi:10.4319/lo.2009.54.4.1210 (2009).
4 Iversen, M. H. *et al.* Sinkers or floaters? Contribution from salp pellets to the export flux during a large bloom event in the Southern Ocean. *Deep-Sea Res. Part II* **138**, 116-125, doi:10.1016/j.dsr2.2016.12.004 (2017).
