# Supplementary material for: Salp blooms drive strong increases in passive carbon export in the Southern Ocean
Source: Nat Commun. 2023 Feb 2;14:425. doi: 10.1038/s41467-022-35204-6 (PMC9894854; doi:10.1038/s41467-022-35204-6)
Supplement: Supplementary file 2 — Reporting Summary [file 41467_2022_35204_MOESM2_ESM.pdf]

nature portfolio

Corresponding author(s): mdecima@ucsd.edu

Last updated by author(s): Nov 19, 2022

Reporting Summary

Nature Portfolio wishes to improve the reproducibility of the work that we publish. This form provides structure for consistency and transparency in reporting. For further information on Nature Portfolio policies, see our Editorial Policies and the Editorial Policy Checklist.

Statistics

For all statistical analyses, confirm that the following items are present in the figure legend, table legend, main text, or Methods section.

n/a	Confirmed	
☐	☒	The exact sample size (n) for each experimental group/condition, given as a discrete number and unit of measurement
☐	☒	A statement on whether measurements were taken from distinct samples or whether the same sample was measured repeatedly
☒	☐	The statistical test(s) used AND whether they are one- or two-sided<br>*Only common tests should be described solely by name; describe more complex techniques in the Methods section.*
☒	☐	A description of all covariates tested
☐	☒	A description of any assumptions or corrections, such as tests of normality and adjustment for multiple comparisons
☐	☒	A full description of the statistical parameters including central tendency (e.g. means) or other basic estimates (e.g. regression coefficient) AND variation (e.g. standard deviation) or associated estimates of uncertainty (e.g. confidence intervals)
☒	☐	For null hypothesis testing, the test statistic (e.g. F, t, r) with confidence intervals, effect sizes, degrees of freedom and P value noted<br>*Give P values as exact values whenever suitable.*
☒	☐	For Bayesian analysis, information on the choice of priors and Markov chain Monte Carlo settings
☒	☐	For hierarchical and complex designs, identification of the appropriate level for tests and full reporting of outcomes
☒	☐	Estimates of effect sizes (e.g. Cohen's d, Pearson's r), indicating how they were calculated

Our web collection on statistics for biologists contains articles on many of the points above.

Software and code

Policy information about availability of computer code

Data collection	Most of the data in this manuscript comes from biological or chemical samples that were collected and analyzed.
Data analysis	DAta was analyzed using Matlab 2021b and R and R studio (2021.09.1 + 372)

For manuscripts utilizing custom algorithms or software that are central to the research but not yet described in published literature, software must be made available to editors and reviewers. We strongly encourage code deposition in a community repository (e.g. GitHub). See the Nature Portfolio guidelines for submitting code & software for further information.

Data

Policy information about availability of data

All manuscripts must include a data availability statement. This statement should provide the following information, where applicable:

- Accession codes, unique identifiers, or web links for publicly available datasets
- A description of any restrictions on data availability
- For clinical datasets or third party data, please ensure that the statement adheres to our policy

Data availability: Export flux data and 238U:234Th are deposited in BCO-DMO. DOI for Sediment trap data (doi:10.26008/1912/bco-dmo.813759.1; doi:10.26008/1912/bco-dmo.813859.1; doi:10.26008/1912/bco-dmo.813828.1). DOI for Thorium data is forthcoming. Both will be available under the project 754878.

Salp abundance, salp gut pigments, chl a, size-fractionated chl a, phytoplankton physiology, integrated HPLC, and phytoplankton growth and grazing has been deposited in PANGAEA. DOI will be available in the next week

All bioinformatics data have been deposited in NCBI (16S data – PRJNA670059 and 18S (CTD and Traps) – PRJNA670061). The remaining datasets presented in the manuscript are available in the tables.

Human research participants

Policy information about studies involving human research participants and Sex and Gender in Research.

Reporting on sex and gender	*Use the terms sex (biological attribute) and gender (shaped by social and cultural circumstances) carefully in order to avoid confusing both terms. Indicate if findings apply to only one sex or gender; describe whether sex and gender were considered in study design whether sex and/or gender was determined based on self-reporting or assigned and methods used. Provide in the source data disaggregated sex and gender data where this information has been collected, and consent has been obtained for sharing of individual-level data; provide overall numbers in this Reporting Summary. Please state if this information has not been collected. Report sex- and gender-based analyses where performed, justify reasons for lack of sex- and gender-based analysis.*
Population characteristics	*Describe the covariate-relevant population characteristics of the human research participants (e.g. age, genotypic information, past and current diagnosis and treatment categories). If you filled out the behavioural & social sciences study design questions and have nothing to add here, write "See above."*
Recruitment	*Describe how participants were recruited. Outline any potential self-selection bias or other biases that may be present and how these are likely to impact results.*
Ethics oversight	*Identify the organization(s) that approved the study protocol.*

Note that full information on the approval of the study protocol must also be provided in the manuscript.

Field-specific reporting

Please select the one below that is the best fit for your research. If you are not sure, read the appropriate sections before making your selection.

☐ Life sciences ☐ Behavioural & social sciences ☒ Ecological, evolutionary & environmental sciences

For a reference copy of the document with all sections, see nature.com/documents/nr-reporting-summary-flat.pdf

Ecological, evolutionary & environmental sciences study design

All studies must disclose on these points even when the disclosure is negative.

Study description	Oceanographic cruise, 30 days, multiple types of sampling (nets, CTD, sensors)
Research sample	Salp assemblage as the target zooplankton group to be studied, rest of zooplankton as biomass. Microplankton (chl a, sequences) for identification of microbial community composition
Sampling strategy	Plankton communities are sampled by established protocols that determine depth of sampling and timing of a tow, volumes of water to be filtered, all to enable sufficient biomass/abundance it included in the sample.
Data collection	Nets were led by MD, CTD and water sampling was led by AGR and MRS, PIT was let by MRS, Thorium was led by MRS and TSK, phytoplankton msampling was led by AGR and KS
Timing and spatial scale	Start: October 24th 2018; end November 18th 2018. Sampling was done at least twice daily for zooplankton (to account for diel vertical migration), daily for or twice per cycle for phytoplankton parameters to provide repeatability, once per cycle for PIT samples.
Data exclusions	None
Reproducibility	Results from this study are all field-measurements, including incubations which are technically experimental set-ups. However, the reproducibility does not apply given that the results are dependent on the field conditions when and where the measurements and incubations were carried out
Randomization	N/A
Blinding	N/A

Did the study involve field work? ☒ Yes ☐ No

Field work, collection and transport

Field conditions	Oceanographic cruise, 30 days, multiple types of sampling (nets, CTD, sensors). Late spring early summer conditions of austral hemisphere.
Location	Chatham Rise, New Zealand. Start lat-44.1953; lon: 172.9333; end -45.48616667 lon 179.6671667
Access & import/export	All required Biosecurity permissions required by New Zealand law were complied by through NIWA permits and overseen by NIWA staff
Disturbance	N/A

Reporting for specific materials, systems and methods

We require information from authors about some types of materials, experimental systems and methods used in many studies. Here, indicate whether each material, system or method listed is relevant to your study. If you are not sure if a list item applies to your research, read the appropriate section before selecting a response.

Materials & experimental systems

n/a	Involved in the study
☒	☐ Antibodies
☒	☐ Eukaryotic cell lines
☒	☐ Palaeontology and archaeology
☒	☐ Animals and other organisms
☒	☐ Clinical data
☒	☐ Dual use research of concern

Methods

n/a	Involved in the study
☒	☐ ChIP-seq
☒	☐ Flow cytometry
☒	☐ MRI-based neuroimaging
